# Organ-Specific Phenolic Profiles, Antioxidant Capacity, and Enzyme-Inhibitory Activities of the Turkish Endemic *Origanum bilgeri*

**DOI:** 10.3390/ph19091429

**Published:** 2026-09-10

**Authors:** Zeyneb Karakus, Cengiz Sarikurkcu

**Affiliations:** 1 Chemical Technology Program, Çay Vocational School, Afyon Kocatepe University, Afyonkarahisar 03200, Türkiye; 2 Department of Analytical Chemistry, Faculty of Pharmacy, Afyonkarahisar Health Sciences University, Afyonkarahisar 03100, Türkiye

**Keywords:** *Origanum bilgeri*, organ-specific phenolic profile, ultrasound-assisted extraction, LC–MS/MS, antioxidant capacity, relative antioxidant capacity index, α-glucosidase inhibition, rosmarinic acid, verbascoside

## Abstract

**Background/Objectives:** *Origanum bilgeri* P.H. Davis is a Turkish endemic Lamiaceae species whose non-volatile phenolic composition and organ-specific bioactivities remain unexplored. This study investigated the phenolic composition of flower, leaf, stem, and root extracts and evaluated their antioxidant and enzyme-inhibitory activities. **Methods:** Ultrasound-assisted ethanolic extracts of the four organs were characterized by liquid chromatography–tandem mass spectrometry (LC–MS/MS) targeting 28 phenolic compounds. Total phenolic and flavonoid contents, outcomes of six antioxidant assays, and inhibitory activities against acetylcholinesterase, butyrylcholinesterase, tyrosinase, α-amylase, and α-glucosidase were determined. Measurements were performed in triplicate as technical replicates, and organ-level differences were interpreted descriptively. The relative antioxidant capacity index (RACI) was calculated from the standardized outcomes of the six direct antioxidant assays, with total phenolic content (TPC) and total flavonoid content (TFC) excluded from the index. Pearson correlation analysis and principal component analysis (PCA) were used for exploratory evaluation of phytochemical and bioactivity patterns. **Results:** Twenty phenolic compounds were detected in at least one organ. Rosmarinic acid predominated in all organs and was most abundant in the leaf (3236 µg/g extract) and stem (3093 µg/g extract), whereas the root was markedly enriched in verbascoside (2775 µg/g extract) and chlorogenic acid (1461 µg/g extract) and exhibited the highest total phenolic content, reaching 160.87 mg gallic acid equivalents (GAE)/g extract. The assay-based RACI ranked the root highest (0.54), followed by the stem (0.02), leaf (−0.27), and flower (−0.30). The root also showed the strongest 2,2-diphenyl-1-picrylhydrazyl (DPPH) radical scavenging activity (half-maximal inhibitory concentration, IC_50_ = 0.28 mg/mL) and cupric reducing antioxidant capacity (CUPRAC) (half-maximal effective concentration, EC_50_ = 0.22 mg/mL) among the investigated organ extracts. Under the present assay conditions, all organ extracts yielded lower α-glucosidase IC_50_ values (0.97–1.10 mg/mL) than acarbose (1.16 mg/mL). Exploratory Pearson correlation analysis indicated strong organ-level covariation among several antioxidant parameters, phenolic constituents, and α-glucosidase inhibitory activity. PCA based on the Pearson correlation matrix showed that principal components 1 and 2 (PC1 and PC2) accounted for 50.73% and 38.49% of the total variance, respectively (cumulative variance: 89.22%), providing an exploratory visualization of the multivariate organization of phytochemical and bioactivity variables. **Conclusions:** *O. bilgeri* exhibited distinct organ-specific phytochemical and bioactivity patterns under the conditions investigated. The root was particularly characterized by high verbascoside and chlorogenic acid contents, elevated total phenolic content, strong antioxidant performance across several assays, and α-glucosidase inhibitory activity. These findings support further investigation of *O. bilgeri*, particularly its root, using independent biological replicates and complementary phytochemical and biological approaches.

## 1. Introduction

Phenolic compounds constitute one of the most extensively studied classes of plant secondary metabolites, owing to their structural diversity and multifaceted biological activities [1]. These compounds exert antioxidant effects through mechanisms including hydrogen atom transfer and single-electron transfer and can also inhibit biologically relevant enzymes such as acetylcholinesterase, tyrosinase, α-amylase, and α-glucosidase [2,3]. The distribution of specialized metabolites within a plant is not uniform; their biosynthesis and accumulation are often organ- and tissue-specific and are regulated by developmental, biotic and abiotic environmental factors [1,4,5]. This spatial variation highlights the importance of organ-level characterization when assessing the phytochemical and pharmacological potential of medicinal plants. Comparative profiling of individual organs can reveal metabolically specialized and biologically active plant parts and may provide a scientific basis for selecting plant material for further phytochemical and pharmacological investigation [6,7].

The genus *Origanum* L. (Lamiaceae) comprises approximately 43 species and 18 hybrids, with Turkey representing a major center of diversity harboring more than 30 taxa, many of which are endemic [8]. While the essential oil chemistry of *Origanum* species has been extensively studied, attention to non-volatile phenolic constituents—particularly rosmarinic acid, salvianolic acids, and flavonoids—has grown considerably in recent years [9,10]. Liquid chromatography–tandem mass spectrometry (LC–MS/MS)-based profiling has identified rosmarinic acid as the predominant phenolic compound across multiple *Origanum* taxa, accompanied by caffeic acid, luteolin, apigenin, and their glycosides [11,12]. Comparative studies have further demonstrated substantial interspecific variation: among six species of the section Brevifilamentum, *O. acutidens* showed the highest antioxidant and anticholinesterase activities [13], while Cretan *Origanum* species exhibited species-specific polyphenolic fingerprints [12]. Nonetheless, investigations addressing non-volatile phenolic composition at the organ level remain limited within the genus, and organ-specific profiles of individual phenolic compounds have been documented in only a few taxa.

*Origanum bilgeri* P.H. Davis is a Turkish endemic species with a restricted distribution in Antalya Province in southwestern Turkey. Previous phytochemical investigations of *O. bilgeri* have focused predominantly on its essential oil composition and associated biological activities, including antibacterial effects against agricultural pathogens [14,15] and acaricidal activity attributed to its major volatile component carvacrol [16]. To the best of our knowledge, however, the non-volatile phenolic composition of *O. bilgeri* has not been characterized, and no study has systematically compared the phenolic and bioactivity profiles of its individual organs. This absence leaves fundamental questions unanswered regarding the species’ phenolic diversity, the distribution of bioactive compounds among tissues, antioxidant capacity, and enzyme-inhibitory potential. The research gap is particularly notable given that phenolic compounds contribute substantially to the antioxidant and enzyme-inhibitory activities observed in other *Origanum* species [17,18,19,20].

Because plant organs differ in their physiological functions and environmental exposure, specialized metabolites are often distributed unevenly among tissues [4,5]. Evidence of such organ-dependent variation has also been reported within *Origanum*. In *O. majorana*, phenolic contents differed substantially among leaves, stems, roots, and flowers, accompanied by differences in antioxidant and antimicrobial activities [21]. Similarly, in *Origanum vulgare*, rhizomes and leaves exhibited distinct phenolic compositions, with salvianolic acid A detected exclusively in rhizomes [18]. These observations indicate that organ-level analysis can uncover phytochemical diversity and functional specialization that whole-plant or aerial-part-only studies would obscure.

Accordingly, this study aimed to characterize the organ-specific phenolic composition and bioactivity profiles of *O. bilgeri* flower, leaf, stem, and root extracts obtained by ultrasound-assisted ethanol extraction. Specifically, we aimed to (i) characterize phenolic profiles across the four organs by LC–MS/MS; (ii) evaluate antioxidant capacity using complementary assays representing different antioxidant mechanisms, including 2,2-diphenyl-1-picrylhydrazyl (DPPH), 2,2′-azino-bis(3-ethylbenzothiazoline-6-sulfonic acid) (ABTS), cupric reducing antioxidant capacity (CUPRAC), ferric reducing antioxidant power (FRAP)**,** phosphomolybdenum, and metal chelating assays; (iii) determine inhibitory activities against acetylcholinesterase (AChE), butyrylcholinesterase (BChE), tyrosinase, α-amylase, and α-glucosidase; and (iv) summarize the six direct antioxidant assay outcomes using the relative antioxidant capacity index (RACI) and explore organ-specific patterns of covariation among phytochemical and bioactivity variables using Pearson correlation analysis and principal component analysis (PCA).

## 2. Results

### 2.1. Total Phenolic and Flavonoid Contents

**Figure 1:**
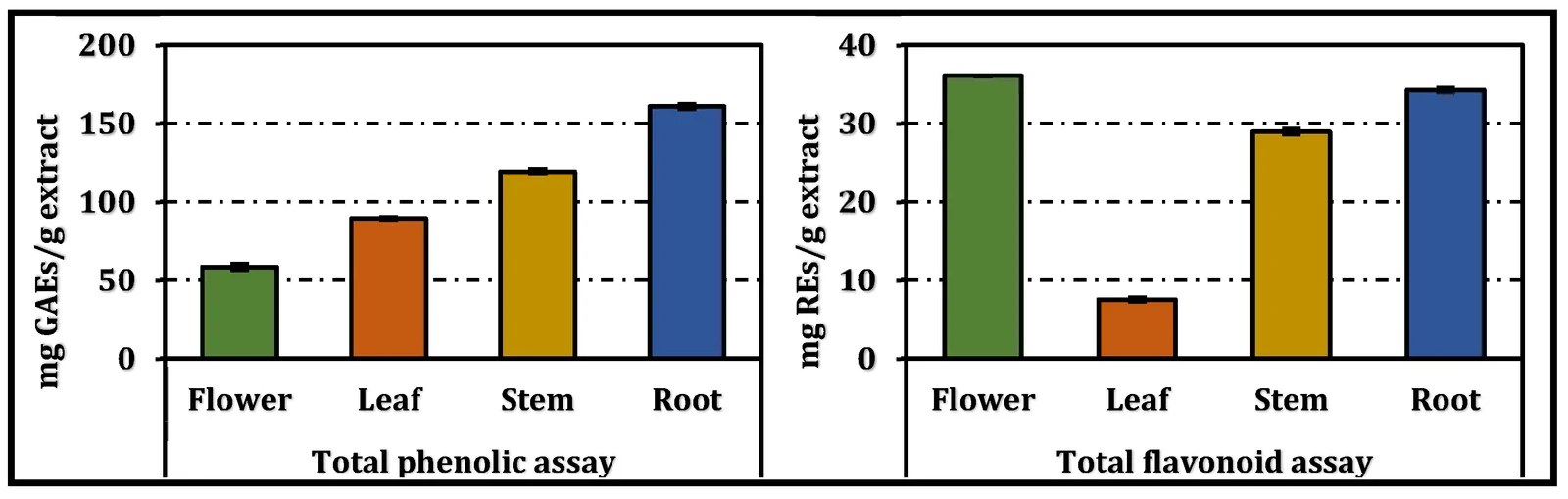
Total phenolic and total flavonoid contents of ultrasound-assisted ethanolic extracts obtained from different organs of *O. bilgeri*. Data are expressed as the mean ± standard deviation (SD) of three technical replicate measurements for each composite organ extract. Error bars represent the variability among technical replicate measurements and therefore reflect analytical repeatability rather than biological variation among individual plants. Because each organ was represented by a single composite extract, no inferential statistical comparisons were performed among organs. Total phenolic content is expressed as mg gallic acid equivalents (GAE)/g extract, and total flavonoid content is expressed as mg rutin equivalents (RE)/g extract.

The root extract exhibited the highest total phenolic content (TPC; 160.87 ± 1.44 mg GAE/g extract), followed by the stem (119.26 ± 1.49 mg GAE/g extract), leaf (89.47 ± 0.70 mg GAE/g extract), and flower (58.43 ± 1.86 mg GAE/g extract) (Figure 1). Thus, TPC values followed the descriptive order root > stem > leaf > flower. In contrast, total flavonoid content (TFC) showed a different organ-dependent distribution, with the highest value observed in the flower (36.07 ± 0.04 mg RE/g extract), followed by the root (34.27 ± 0.25 mg RE/g extract), stem (28.93 ± 0.33 mg RE/g extract), and leaf (7.52 ± 0.25 mg RE/g extract). Accordingly, TFC values followed the descriptive order flower > root > stem > leaf. Because TPC values are expressed as gallic acid equivalents, they represent an equivalence-based Folin–Ciocalteu response rather than a direct measurement of the absolute mass of phenolic compounds in the extracts. Accordingly, these values should not be directly compared on a mass basis with the concentrations of individual compounds quantified by targeted LC–MS/MS.

### 2.2. LC–MS/MS Phenolic Profiling

Of the 28 phenolic compounds targeted, 20 were detected in at least one organ extract, whereas 8 compounds ((−)-epicatechin, (+)-catechin, 2-hydroxycinnamic acid, hesperidin, kaempferol, pinoresinol, sinapic acid, and syringic acid) were not detected in any organ (Table 1).

Rosmarinic acid was the predominant phenolic compound in all four organs, with the highest concentrations in the leaf (3236 ± 2 µg/g extract) and stem (3093 ± 39 µg/g extract), which showed similar values, followed by the flower (2776 ± 29 µg/g extract) and root (1500 ± 12 µg/g extract).

Flower and leaf extracts were characterized by relatively high concentrations of flavonoid aglycones. Eriodictyol occurred at similar concentrations in the leaf (906 ± 9 µg/g extract) and flower (889 ± 9 µg/g extract), but at substantially lower concentrations in the stem (113 ± 1 µg/g extract) and root (6.51 ± 0.10 µg/g extract).

Apigenin followed the order flower (238 ± 2 µg/g extract) > leaf (220 ± 1 µg/g extract) > stem (27.6 ± 0.1 µg/g extract) > root (1.90 ± 0.05 µg/g extract), with a clear descriptive gradient among the organs. Taxifolin was detected in the flower (225 ± 1 µg/g extract), leaf (203 ± 1 µg/g extract), and stem (11.0 ± 0.2 µg/g extract), but was not detected in the root. Luteolin and quercetin were likewise detected only in aerial organs, with their highest concentrations observed in the leaf (96.8 ± 1.0 and 57.7 ± 1.6 µg/g extract, respectively).

The root extract exhibited a markedly different phenolic profile, characterized by pronounced enrichment of verbascoside and chlorogenic acid. Verbascoside reached 2775 ± 55 µg/g extract in the root, more than 600-fold higher than in the aerial organs (2.37–4.14 µg/g extract). Chlorogenic acid accumulated to 1461 ± 22 µg/g extract in the root, approximately 51-fold higher than in the stem (28.6 ± 0.4 µg/g extract) and approximately 140- and 209-fold higher than in the flower and leaf, respectively.

The stem extract contained the highest concentrations of several phenolic acids and related compounds, including caffeic acid (354 ± 1 µg/g extract), protocatechuic acid (198 ± 2 µg/g extract), 3,4-dihydroxyphenylacetic acid (91.6 ± 0.2 µg/g extract), vanillin (125 ± 5 µg/g extract), 3-hydroxybenzoic acid (23.8 ± 1.0 µg/g extract), and 4-hydroxybenzoic acid (23.8 ± 1.5 µg/g extract), with all of these compounds showing their highest observed concentrations in the stem among the investigated organs.

### 2.3. Antioxidant Activities

**Figure 2:**
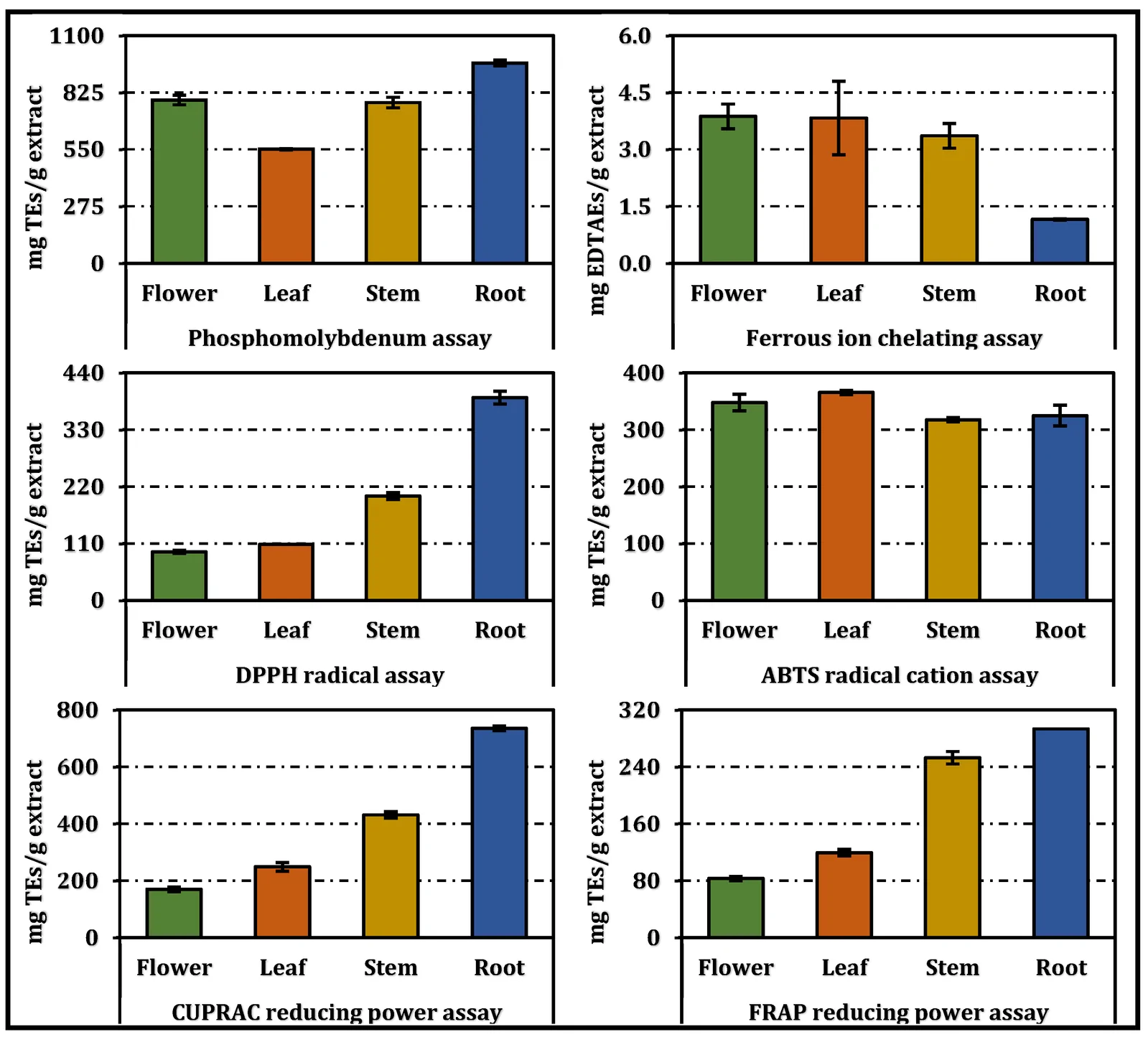
The antioxidant activities of ultrasound-assisted ethanolic extracts obtained from different organs of *O. bilgeri*. Data are expressed as the mean ± standard deviation (SD) of three technical replicate measurements for each composite organ extract. Error bars represent the variability among technical replicate measurements and therefore reflect analytical repeatability rather than biological variation among individual plants. Because each organ was represented by a single composite extract, no inferential statistical comparisons were performed among organs. Antioxidant activities are expressed as mg Trolox equivalents (TE)/g extract and mg EDTA equivalents (EDTAE)/g extract.

The antioxidant activities of the organ-specific ethanolic extracts were evaluated using six complementary assays. The results are presented as IC_50_ values for the DPPH, ABTS, and metal chelating assays and as EC_50_ values for the CUPRAC, FRAP, and phosphomolybdenum assays (Table 2), together with the corresponding standard-equivalent antioxidant capacities (Figure 2).

#### 2.3.1. Radical Scavenging Assays

DPPH radical scavenging activity showed a clear descriptive variation across the four organs. Root extract showed the strongest activity, with the highest Trolox-equivalent value (392.12 ± 12.53 mg TE/g extract) and the lowest IC_50_ value (0.28 ± 0.009 mg/mL), followed by the stem (201.79 ± 6.70 mg TE/g extract; IC_50_ = 0.54 ± 0.018 mg/mL). The leaf and flower exhibited lower DPPH activities, with equivalent values of 108.27 ± 0.29 and 94.06 ± 2.91 mg TE/g extract, respectively. Their Trolox-equivalent values were similar in magnitude, whereas their IC_50_ values were 1.01 ± 0.003 and 1.17 ± 0.036 mg/mL, respectively. The Trolox standard exhibited an IC_50_ value of 0.11 ± 0.007 mg/mL.

ABTS radical scavenging activity was comparatively similar among the organs. Trolox-equivalent values ranged from 317.48 ± 3.86 to 365.35 ± 3.41 mg TE/g extract, indicating a relatively narrow range across the flower, leaf, stem, and root. The corresponding IC_50_ values ranged from 0.28 ± 0.003 to 0.32 ± 0.004 mg/mL. The leaf showed the lowest IC_50_ value (0.28 ± 0.003 mg/mL), followed by the flower (0.29 ± 0.012 mg/mL), root (0.31 ± 0.018 mg/mL), and stem (0.32 ± 0.004 mg/mL). Trolox exhibited the lowest IC_50_ value (0.10 ± 0.004 mg/mL).

#### 2.3.2. Reducing Capacity Assays

CUPRAC reducing capacity showed a clear descriptive gradient across the four organs. The root extract exhibited the highest value (735.47 ± 8.65 mg TE/g extract), followed by the stem (430.80 ± 11.19 mg TE/g extract), leaf (248.44 ± 15.26 mg TE/g extract), and flower (169.72 ± 7.63 mg TE/g extract). Consistently, the root showed the lowest EC_50_ value (0.22 ± 0.003 mg/mL), followed by the stem (0.38 ± 0.010 mg/mL), leaf (0.65 ± 0.040 mg/mL), and flower (0.96 ± 0.043 mg/mL). The root EC_50_ value was close in magnitude to that of Trolox (0.16 ± 0.011 mg/mL).

FRAP reducing capacity followed a similar organ-dependent pattern, with the highest value observed in the root (293.29 ± 0.10 mg TE/g extract), followed by the stem (252.73 ± 8.75 mg TE/g extract), leaf (119.39 ± 4.33 mg TE/g extract), and flower (82.96 ± 2.97 mg TE/g extract). The root and stem showed the two highest Trolox-equivalent values, whereas the leaf and flower showed lower values. Similarly, the EC_50_ values were lowest for the root (0.16 ± 0.001 mg/mL) and stem (0.19 ± 0.007 mg/mL), followed by the leaf (0.40 ± 0.015 mg/mL) and flower (0.58 ± 0.021 mg/mL). Trolox exhibited an EC_50_ value of 0.05 ± 0.003 mg/mL.

In the phosphomolybdenum assay, the root exhibited the highest total antioxidant capacity (967.00 ± 13.95 mg TE/g extract), followed by the flower (788.68 ± 23.37 mg TE/g extract) and stem (776.85 ± 25.46 mg TE/g extract), which showed similar values, while the leaf showed the lowest value (550.92 ± 3.14 mg TE/g extract). The root EC_50_ value (0.35 ± 0.005 mg/mL) was close to that of Trolox (0.33 ± 0.007 mg/mL), whereas the flower and stem showed nearly identical EC_50_ values (0.44 ± 0.013 and 0.44 ± 0.014 mg/mL, respectively).

#### 2.3.3. Metal Chelating Activity

Metal chelating activity showed a distinct organ-dependent pattern. The flower (3.87 ± 0.33 mg EDTAE/g extract), leaf (3.83 ± 0.97 mg EDTAE/g extract), and stem (3.36 ± 0.33 mg EDTAE/g extract) exhibited similar activities, whereas the root showed a lower value (1.16 ± 0.02 mg EDTAE/g extract). Consistently, the IC_50_ values of the flower (5.21 ± 0.439 mg/mL), leaf (5.43 ± 1.374 mg/mL), and stem (6.01 ± 0.583 mg/mL) were relatively similar in magnitude, while the root exhibited a higher IC_50_ value (17.38 ± 0.27 mg/mL), indicating the weakest metal chelating activity among the investigated organs. EDTA showed an IC_50_ value of 0.02 ± 0.001 mg/mL and was more active than the organ extracts under the present assay conditions.

#### 2.3.4. Relative Antioxidant Capacity Index (RACI)

**Figure 3:**
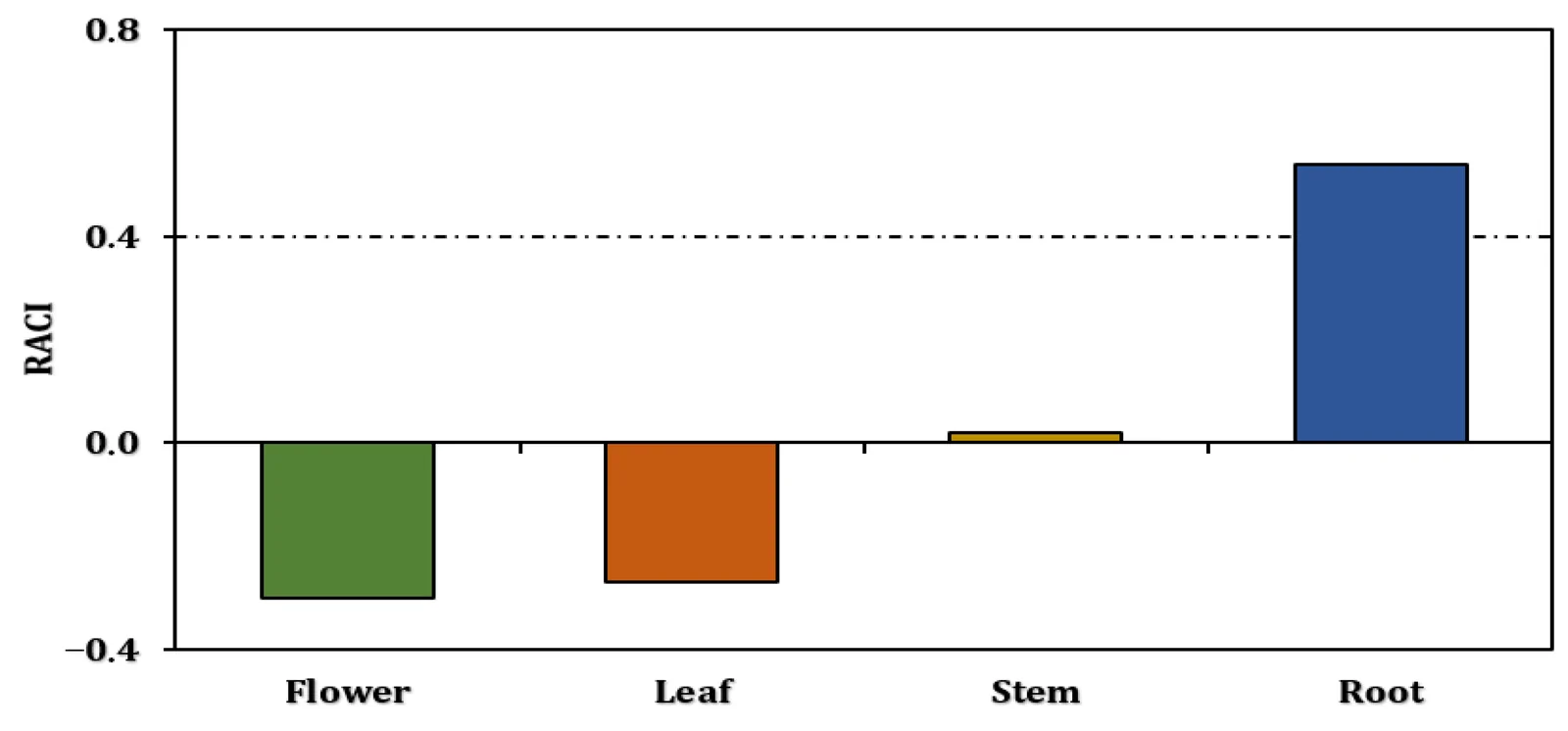
The relative antioxidant capacity index (RACI) values of ultrasound-assisted ethanolic extracts obtained from different organs of *O. bilgeri*. The RACI was calculated by standardizing and integrating the results of six direct antioxidant assays, namely phosphomolybdenum, DPPH, ABTS, CUPRAC, FRAP, and metal chelating assays. Higher RACI values indicate greater relative assay-integrated antioxidant performance across the evaluated methods. Total phenolic content (TPC) and total flavonoid content (TFC) were not included in the RACI calculation, thereby restricting the index to direct antioxidant assay outcomes. As each organ was represented by a single composite extract, the RACI values are presented descriptively and are not intended for inferential comparison among organs.

The relative antioxidant capacity index (RACI) was calculated from the standardized outcomes of the six direct antioxidant assays and showed a distinct descriptive pattern among the investigated organs (Figure 3). The root exhibited the highest RACI value (0.54), indicating the greatest relative assay-integrated antioxidant performance, followed by the stem (0.02). The leaf and flower showed negative RACI values of −0.27 and −0.30, respectively. Accordingly, the assay-based RACI followed the descriptive order root > stem > leaf > flower.

Notably, exclusion of TPC and TFC from the RACI calculation retained the root and stem as the two highest ranked organs, although the relative order of leaf and flower was reversed. Thus, the predominance of the root in the assay-integrated antioxidant assessment was maintained when the index was restricted to direct antioxidant assay outcomes.

### 2.4. Enzyme-Inhibitory Activities

**Figure 4:**
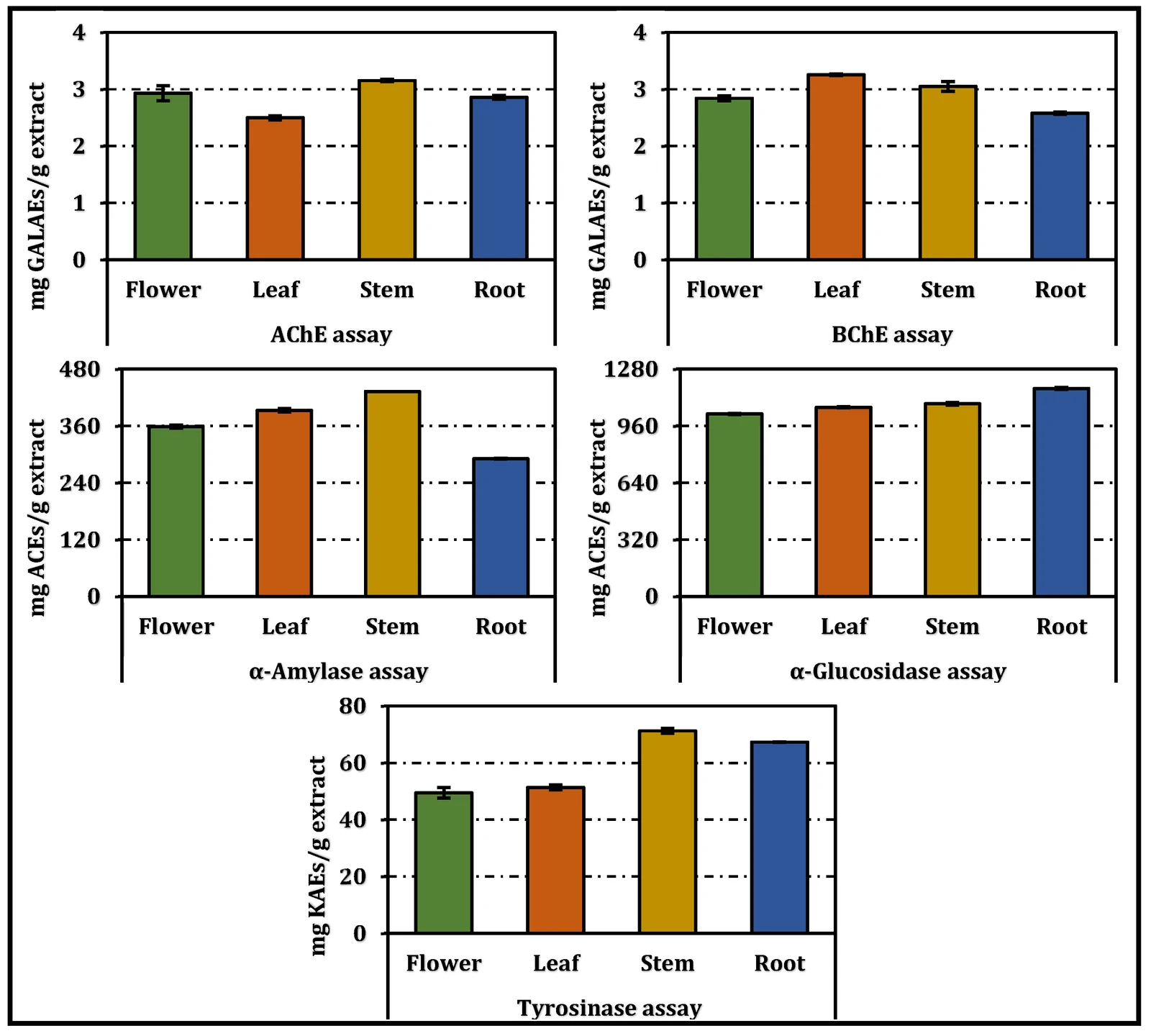
The enzyme-inhibitory activities of ultrasound-assisted ethanolic extracts obtained from different organs of *O. bilgeri*. Data are expressed as the mean ± standard deviation (SD) of three technical replicate measurements for each composite organ extract. Error bars represent the variability among technical replicate measurements and therefore reflect analytical repeatability rather than biological variation among individual plants. Because each organ was represented by a single composite extract, no inferential statistical comparisons were performed among organs. Enzyme-inhibitory activities are expressed as mg galantamine equivalents (GALAE)/g extract, mg kojic acid equivalents (KAE)/g extract, and mg acarbose equivalents (ACE)/g extract.

The enzyme-inhibitory activities of the organ-specific ethanolic extracts were evaluated against acetylcholinesterase (AChE), butyrylcholinesterase (BChE), tyrosinase, α-amylase, and α-glucosidase. The results are presented as IC_50_ values (Table 3), with lower values indicating stronger inhibitory activity, while the corresponding positive-control-equivalent activities are presented in Figure 4.

#### 2.4.1. Cholinesterases

Acetylcholinesterase (AChE) inhibition showed an organ-dependent pattern. The stem exhibited the lowest IC_50_ value (1.02 ± 0.007 mg/mL), followed by the flower (1.09 ± 0.050 mg/mL), root (1.12 ± 0.014 mg/mL), and leaf (1.28 ± 0.019 mg/mL). The IC_50_ values of the stem, flower, and root were relatively close in magnitude, whereas the leaf showed the highest IC_50_ value among the organ extracts.

Butyrylcholinesterase (BChE) inhibition was strongest in the leaf (IC_50_ = 0.98 ± 0.004 mg/mL), followed by the stem (1.05 ± 0.029 mg/mL), flower (1.13 ± 0.017 mg/mL), and root (1.24 ± 0.010 mg/mL), yielding the descriptive order leaf > stem > flower > root in inhibitory activity. Galantamine exhibited a markedly lower IC_50_ value (0.0032 ± 0.0002 mg/mL) than all organ extracts.

#### 2.4.2. Tyrosinase

Tyrosinase inhibitory activity showed an organ-dependent pattern. The stem and root exhibited the lowest IC_50_ values (1.16 ± 0.015 and 1.23 ± 0.002 mg/mL, respectively), with relatively similar values. Both extracts showed stronger inhibition than the leaf (1.61 ± 0.027 mg/mL) and flower (1.67 ± 0.064 mg/mL), which also showed similar IC_50_ values. The corresponding kojic acid-equivalent activities showed the same organ-dependent pattern. Kojic acid exhibited a lower IC_50_ value (0.08 ± 0.002 mg/mL) than all organ extracts.

#### 2.4.3. Carbohydrate Hydrolyzing Enzymes

α-Amylase inhibitory activity showed a clear descriptive variation among the four organ extracts. The stem exhibited the lowest IC_50_ value (2.17 ± 0.002 mg/mL), followed by the leaf (2.39 ± 0.021 mg/mL), flower (2.62 ± 0.022 mg/mL), and root (3.23 ± 0.010 mg/mL). All organ extracts showed higher IC_50_ values than acarbose (0.94 ± 0.035 mg/mL), indicating weaker α-amylase inhibition under the present assay conditions. The corresponding acarbose-equivalent activities showed the same organ-dependent pattern (Figure 4).

In contrast, all four organ extracts exhibited lower α-glucosidase IC_50_ values than acarbose (1.16 ± 0.026 mg/mL) under the present assay conditions. The root showed the strongest inhibition (IC_50_ = 0.97 ± 0.004 mg/mL), followed by the stem (1.05 ± 0.007 mg/mL), leaf (1.06 ± 0.002 mg/mL), and flower (1.10 ± 0.003 mg/mL). The IC_50_ values of the stem, leaf, and flower were relatively close in magnitude, while the root showed the lowest IC_50_ value among the organ extracts.

### 2.5. Pearson Correlation Analysis

**Figure 5:**
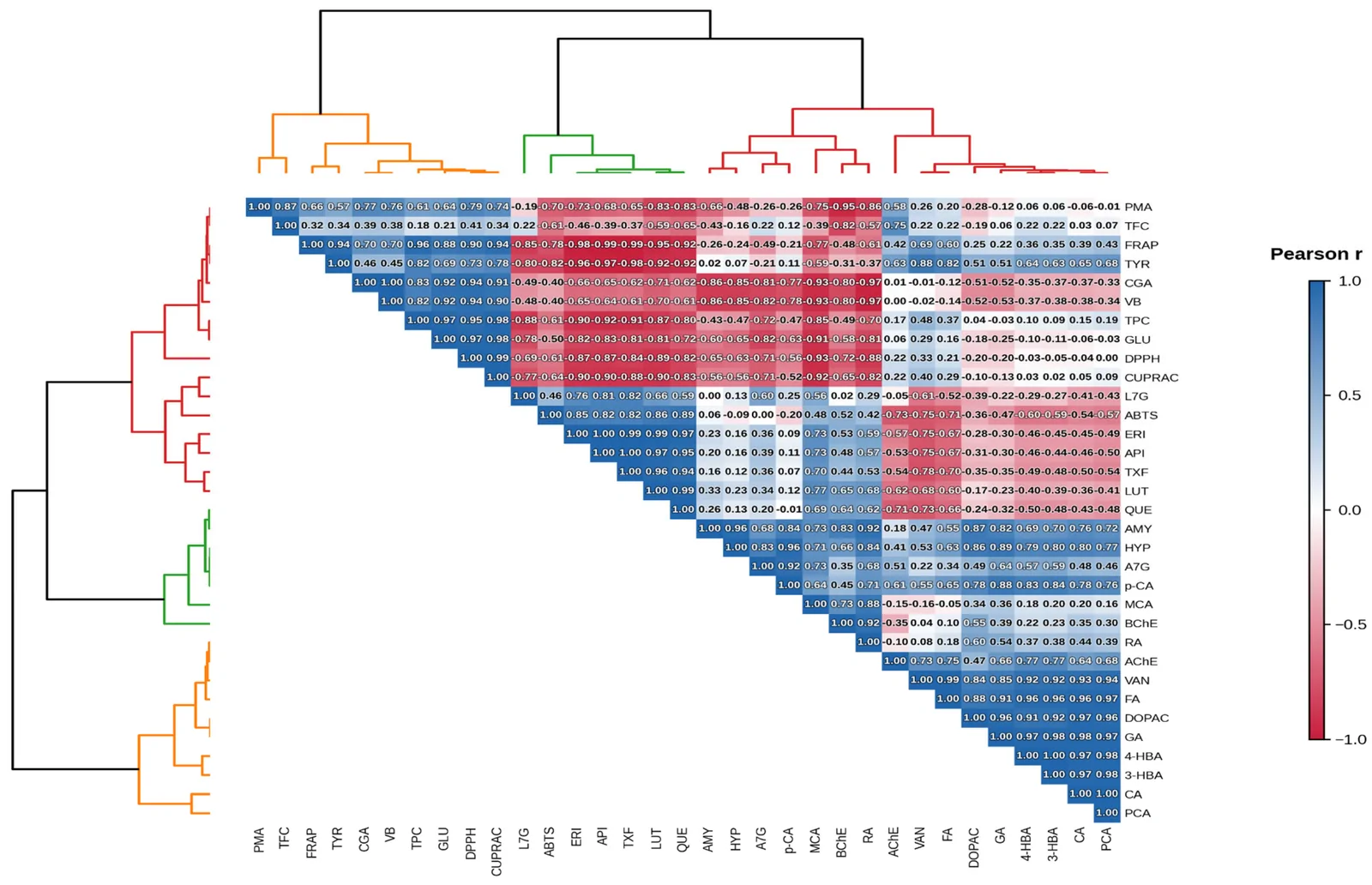
A hierarchically clustered Pearson correlation heatmap showing the patterns of covariation among phytochemical parameters, antioxidant activities, enzyme-inhibitory activities, and quantified phenolic compounds of ultrasound-assisted ethanolic extracts obtained from different organs of *O. bilgeri*. The correlation matrix was calculated using the mean values of four composite organ extracts (*n* = 4 organ-level observations) and is presented as an exploratory visualization rather than for confirmatory statistical inference. Pearson correlation coefficients (r) are displayed within the cells; blue indicates positive correlations, red indicates negative correlations, and white corresponds to r = 0. Only the upper triangular matrix, including the diagonal, is shown. Hierarchical clustering of rows and columns was performed using UPGMA (average linkage) with 1 − r as the distance measure. PMA, phosphomolybdenum assay; DPPH, 2,2-diphenyl-1-picrylhydrazyl radical scavenging assay; ABTS, 2,2′-azino-bis(3-ethylbenzothiazoline-6-sulfonic acid) radical scavenging assay; CUPRAC, cupric reducing antioxidant capacity; FRAP, ferric reducing antioxidant power; MCA, metal chelating activity; AChE, acetylcholinesterase inhibition; BChE, butyrylcholinesterase inhibition; TYR, tyrosinase inhibition; AMY, α-amylase inhibition; GLU, α-glucosidase inhibition; TPC, total phenolic content; TFC, total flavonoid content; RA, rosmarinic acid; ERI, eriodictyol; API, apigenin; TXF, taxifolin; A7G, apigenin 7-glucoside; CA, caffeic acid; LUT, luteolin; QUE, quercetin; DOPAC, 3,4-dihydroxyphenylacetic acid; PCA, protocatechuic acid; L7G, luteolin 7-glucoside; VAN, vanillin; FA, ferulic acid; p-CA, p-coumaric acid; CGA, chlorogenic acid; 4-HBA, 4-hydroxybenzoic acid; 3-HBA, 3-hydroxybenzoic acid; HYP, hyperoside; VB, verbascoside; GA, gallic acid.

Pearson correlation analysis was used to explore patterns of covariation among the phytochemical parameters, antioxidant assays, enzyme-inhibitory parameters, and individual phenolic compounds, as visualized by the hierarchically clustered correlation heatmap (Figure 5). The analysis was based on the mean values of the four composite organ extracts (*n* = 4 organ-level observations).

Large positive Pearson correlation coefficients were observed among several antioxidant-related parameters. In particular, DPPH was positively correlated with CUPRAC (r = 0.992), while TPC showed large positive correlation coefficients with CUPRAC (r = 0.980), FRAP (r = 0.958), and DPPH (r = 0.951). The α-glucosidase inhibitory parameter (GLU) also showed large positive correlation coefficients with CUPRAC (r = 0.982), DPPH (r = 0.975), and TPC (r = 0.973). In contrast, MCA displayed large negative correlation coefficients with DPPH (r = −0.933), CUPRAC (r = −0.918), and GLU (r = −0.911).

Several individual phenolic compounds exhibited highly coordinated organ-level variation. Chlorogenic acid (CGA) and verbascoside (VB) showed an almost perfect positive correlation (r = 0.999) and large positive correlation coefficients with DPPH (CGA, r = 0.941; VB, r = 0.936), CUPRAC (CGA, r = 0.905; VB, r = 0.900), and GLU (CGA, r = 0.922; VB, r = 0.918). Large positive correlation coefficients were also observed between apigenin and taxifolin (r = 0.999), caffeic acid and protocatechuic acid (r = 0.998), 4-hydroxybenzoic acid and 3-hydroxybenzoic acid (r = 0.999), luteolin and quercetin (r = 0.991), and vanillin and ferulic acid (r = 0.991). Conversely, FRAP showed large negative correlation coefficients with apigenin (r = −0.992), taxifolin (r = −0.988), and eriodictyol (r = −0.983), whereas tyrosinase inhibition showed similarly large negative correlation coefficients with taxifolin (r = −0.980), apigenin (r = −0.970), and eriodictyol (r = −0.964).

The hierarchical clustering pattern was consistent with these organ-level covariance patterns, producing closely associated subgroups of variables with similar correlation profiles. DPPH, CUPRAC, TPC, GLU, CGA, and VB were positioned within a closely related region of the dendrogram, whereas ABTS clustered more closely with the flavonoid-related variables ERI, API, TXF, LUT, and QUE. A separate phenolic-rich grouping included CA, PCA, 4-HBA, 3-HBA, GA, FA, VAN, and DOPAC, reflecting the similar patterns of variation observed across the four organ extracts (Figure 5).

Because the analysis was based on four organ-level observations (*n* = 4), the correlation coefficients should be interpreted as exploratory indicators of organ-level covariation rather than as statistically generalizable evidence of causal or mechanistic associations.

### 2.6. Principal Component Analysis

**Figure 6:**
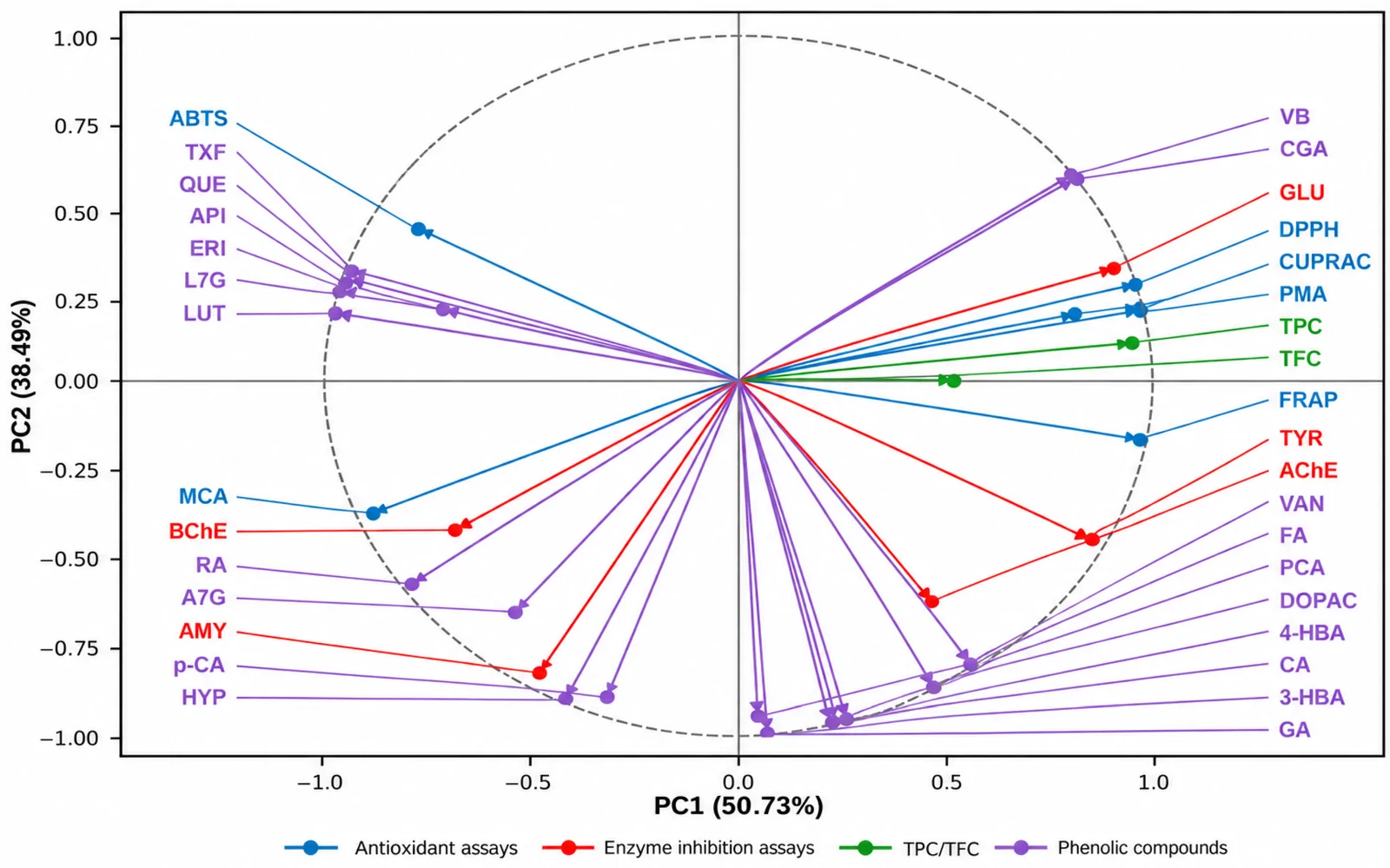
A principal component analysis (PCA) loading plot showing the exploratory multivariate organization of phytochemical parameters, antioxidant activities, enzyme-inhibitory activities, and quantified phenolic compounds of ultrasound-assisted ethanolic extracts obtained from different organs of *O. bilgeri*. The PCA was based on the mean values of four composite organ extracts (*n* = 4 organ-level observations) and is presented as an exploratory visualization rather than for confirmatory statistical inference. PC1 and PC2 accounted for 50.73% and 38.49% of the total variance, respectively, explaining 89.22% of the cumulative variance. PMA, phosphomolybdenum assay; DPPH, 2,2-diphenyl-1-picrylhydrazyl radical scavenging assay; ABTS, 2,2′-azino-bis(3-ethylbenzothiazoline-6-sulfonic acid) radical scavenging assay; CUPRAC, cupric reducing antioxidant capacity; FRAP, ferric reducing antioxidant power; MCA, metal chelating activity; AChE, acetylcholinesterase inhibition; BChE, butyrylcholinesterase inhibition; TYR, tyrosinase inhibition; AMY, α-amylase inhibition; GLU, α-glucosidase inhibition; TPC, total phenolic content; TFC, total flavonoid content; RA, rosmarinic acid; ERI, eriodictyol; API, apigenin; TXF, taxifolin; A7G, apigenin 7-glucoside; CA, caffeic acid; LUT, luteolin; QUE, quercetin; DOPAC, 3,4-dihydroxyphenylacetic acid; PCA, protocatechuic acid; L7G, luteolin 7-glucoside; VAN, vanillin; FA, ferulic acid; p-CA, p-coumaric acid; CGA, chlorogenic acid; 4-HBA, 4-hydroxybenzoic acid; 3-HBA, 3-hydroxybenzoic acid; HYP, hyperoside; VB, verbascoside; GA, gallic acid.

Principal component analysis (PCA) based on the Pearson correlation matrix provided an integrated exploratory representation of the patterns of variation among phytochemical parameters, antioxidant activities, enzyme-inhibitory activities, and individual phenolic compounds (Figure 6). The analysis was based on the mean values of the four composite organ extracts (*n* = 4 organ-level observations). The first two principal components accounted for 50.73% and 38.49% of the total variance, respectively, explaining 89.22% of the cumulative variance.

The PCA loading plot revealed distinct patterns among the investigated variables. DPPH, CUPRAC, FRAP, TPC, α-glucosidase inhibition, chlorogenic acid, and verbascoside were positioned in a similar direction within the PC1–PC2 space, indicating similar patterns of organ-level multivariate variation. Chlorogenic acid and verbascoside were located particularly close to each other, consistent with their similar organ-level variation observed in the Pearson analysis. In contrast, metal chelating activity and several flavonoid-related variables, including eriodictyol, apigenin, taxifolin, luteolin, and quercetin, were oriented in a different region of the loading space.

Other phenolic compounds, including caffeic acid, protocatechuic acid, 4-hydroxybenzoic acid, 3-hydroxybenzoic acid, vanillin, ferulic acid, and 3,4-dihydroxyphenylacetic acid, also exhibited similar loading directions, reflecting their closely related variation across the investigated organs. Overall, the PCA pattern was consistent with the organ-level covariation patterns identified by the hierarchically clustered Pearson correlation analysis and provided an exploratory visualization of the multivariate organization of the phytochemical and bioactivity variables (Figure 6). Given the limited number of organ-level observations, the PCA should be interpreted descriptively and not as confirmatory evidence of generalizable multivariate relationships.

## 3. Discussion

### 3.1. Organ-Dependent Phytochemical Differentiation

The pronounced root > stem > leaf > flower gradient in TPC observed in *O. bilgeri* indicates marked organ-dependent variation in the Folin–Ciocalteu-reactive reducing capacity of the extracts. This pattern contrasts strongly with that reported for *O. majorana*, in which the aerial vegetative organs were substantially richer in total phenolics than the underground and reproductive organs. Ayari et al. [21] reported polyphenol contents of 58.14 and 48.25 mg GAE/g DW in leaves and stems, respectively, compared with only 9.36 mg GAE/g DW in roots and 11.78 mg GAE/g DW in flowers, corresponding overall to an approximately five-fold enrichment in leaves and stems. Organ-dependent differentiation has also been demonstrated in *O. vulgare*, where leaves and rhizomes exhibited distinct phenolic compositions, including the occurrence of salvianolic acid A in the rhizome extract [18]. Together, these findings indicate that the distribution of phenolic constituents among organs is highly species-dependent within the genus *Origanum*.

Importantly, the Folin–Ciocalteu-derived TPC values should not be interpreted as absolute phenolic mass or directly equated with the summed concentrations obtained by targeted LC–MS/MS. The Folin–Ciocalteu assay is an equivalence-based colorimetric method that primarily reflects the reducing capacity of the sample and is not completely specific to phenolic compounds, because other reducing constituents may also react with the reagent [22,23]. In contrast, the present LC–MS/MS analysis quantitatively measured only the predefined phenolic analytes included in the targeted panel. Consequently, the substantially higher numerical TPC value observed for the root (160.87 mg GAE/g extract) relative to the summed mass of the targeted LC–MS/MS analytes does not represent an analytical discrepancy. Rather, the two approaches provide complementary but analytically non-equivalent information, and the compounds quantified by LC–MS/MS should not be considered to account quantitatively for the entire Folin–Ciocalteu-reactive pool.

A different organ hierarchy was observed for TFC in *O. bilgeri*, with the flower showing the highest value, followed by the root, stem, and leaf. Thus, total phenolic and flavonoid accumulation did not vary in parallel among the investigated organs. This pattern also differs from that reported for *O. majorana*, in which flavonoid contents were markedly higher in leaves (12.27 mg CE/g DW) and stems (11.34 mg CE/g DW) than in roots (3.09 mg CE/g DW) and flowers (2.38 mg CE/g DW) [21]. The contrasting distributions observed between the two species further emphasize that organ-dependent allocation of phenolic subclasses is not conserved uniformly across *Origanum* taxa.

The LC–MS/MS profiles of *O. bilgeri* further illustrated this organ-dependent differentiation. The root was markedly enriched in the phenylethanoid glycoside verbascoside and the hydroxycinnamic acid chlorogenic acid, whereas the flower and leaf contained substantially higher concentrations of several flavonoid aglycones, particularly eriodictyol, apigenin, and taxifolin. These findings demonstrate pronounced organ-dependent partitioning of the targeted phenolic compounds in *O. bilgeri* and are consistent with the distinct antioxidant and enzyme-inhibitory profiles observed among the investigated organs.

### 3.2. Rosmarinic Acid-Rich Aerial Organs

Rosmarinic acid was the predominant phenolic compound in all four *O. bilgeri* organs, with the highest concentrations detected in the leaf (3236 µg/g extract) and stem (3093 µg/g extract), which showed similar concentrations. Its predominance is consistent with the phytochemical characteristics reported for other *Origanum* species. In *O. vulgare* accessions, rosmarinic acid was the most abundant phenolic constituent, occurring at 659.6–1646.9 mg/100 g DW, and was positively associated with antioxidant activity [24]. Rosmarinic acid has likewise been identified as a major phenolic constituent of *O. majorana* extracts, together with other caffeic acid-derived metabolites such as salvianolic and lithospermic acids [9,10]. These findings support the prominent contribution of rosmarinic acid to the non-volatile phenolic profile of the genus *Origanum*.

The well-established in vitro antioxidant potential of rosmarinic acid is closely related to its molecular structure. Rosmarinic acid is an ester of caffeic acid and 3,4-dihydroxyphenyllactic acid and therefore contains two catechol moieties capable of participating in hydrogen atom and electron transfer reactions. However, these two catechol moieties should not be assumed to exhibit equivalent redox reactivity. Electrochemical studies have demonstrated two successive oxidation steps for rosmarinic acid, with the first oxidation step associated with the caffeic acid moiety and the second with the 3,4-dihydroxyphenyllactic acid residue [25]. EPR spectroscopy combined with DFT calculations further showed that oxidation can occur at both catechol groups, while the radical species detected depend on factors such as pH and the oxidizing agent [26]. More recent DFT-based kinetic analyses also indicate that the contribution of individual hydroxyl sites and the preferred radical scavenging pathways of rosmarinic acid vary according to its ionization state and reaction medium [27]. The resulting phenoxyl radicals can be stabilized through resonance and intramolecular hydrogen bonding between adjacent ortho-hydroxyl groups, which contributes to stabilization of the radical forms after hydrogen abstraction, as supported by density functional theory (DFT) calculations [28,29]. Experimental oxidation studies have also demonstrated the formation of quinone-type products from rosmarinic acid; however, the two catechol-containing regions should not be considered equally reactive, because the preferred oxidation pathway and site may depend on the reaction conditions [25,30]. The formation of quinone-type oxidation products also warrants toxicological consideration. Quinones can behave as electrophilic Michael acceptors and redox-active species, enabling covalent modification of cellular nucleophiles and redox cycling with the generation of reactive oxygen species. Depending on their chemical structure, concentration, site of formation, and cellular detoxification capacity, these processes may contribute to oxidative stress and cytotoxic effects [31,32]. However, quinone formation should not be regarded as synonymous with toxicity in all biological contexts, because quinone reactivity and biological consequences are strongly context-dependent [31,32]. In the present study, quinone-type oxidation products were not directly identified or quantified, and their toxicological effects were not evaluated; therefore, no conclusion can be drawn regarding their formation or biological significance in the investigated extracts.

However, the chemical reactivity of rosmarinic acid observed in vitro should not be directly equated with its biological efficacy in vivo. Following oral intake, rosmarinic acid is subject to absorption, distribution, metabolism, and excretion processes that can substantially modify its systemic exposure and biological form. Human pharmacokinetic studies have shown that rosmarinic acid undergoes extensive conjugation and methylation, with glucuronidated and/or sulfated forms predominating in plasma and urine, together with metabolites such as caffeic and ferulic acids [33,34]. Accordingly, the concentration and chemical form of rosmarinic acid reaching target tissues may differ markedly from those evaluated in cell-free antioxidant assays. Its in vivo biological effects should therefore be interpreted in the context of bioavailability, metabolic transformation, and the activity of circulating metabolites rather than solely from the antioxidant reactivity of the parent compound [33,34].

Despite these intrinsic antioxidant properties, the organ-level distribution of rosmarinic acid did not parallel the overall antioxidant pattern observed in *O. bilgeri*. The leaf contained the highest rosmarinic acid concentration but showed a negative assay-based RACI value (−0.27), whereas the root contained the lowest rosmarinic acid concentration (1500 µg/g extract) and yielded the highest assay-based RACI value (0.54). Consistent with this distribution, rosmarinic acid showed negative Pearson correlation coefficients at the organ level with DPPH (r = −0.881), CUPRAC (r = −0.821), and FRAP (r = −0.604). Given that these coefficients were calculated from only four organ-level observations, they should be interpreted as exploratory patterns of covariation rather than as evidence of generalizable biological relationships. This apparent discordance should not be interpreted as evidence against the established antioxidant properties of rosmarinic acid; rather, it indicates that the antioxidant performance of the crude extracts cannot be attributed to the concentration of a single constituent. Differences in the abundance of other phenolic compounds, overall extract composition, and potential additive or interactive effects among constituents likely contribute to the organ-dependent antioxidant profiles observed in *O. bilgeri*.

### 3.3. Distinct Root Phytochemical Signature

The root extract exhibited a phytochemical profile qualitatively and quantitatively distinct from those of the aerial organs, characterized particularly by the pronounced accumulation of verbascoside (2775 µg/g extract) and chlorogenic acid (1461 µg/g extract). Verbascoside (acteoside) is a widely distributed phenylethanoid glycoside with well-established antioxidant and other biological properties [35]. A comparable organ-dependent specialization has recently been reported in *O. vulgare*, in which salvianolic acid A reached 18.29 mg/g in rhizomes but was not detected in leaves [18]. The more than 600-fold enrichment of verbascoside in the root of *O. bilgeri*, together with the pronounced accumulation of chlorogenic acid, therefore demonstrates substantial organ-specific distribution of these targeted phenolic constituents, although the biosynthetic and regulatory mechanisms underlying this distribution remain to be elucidated.

The co-accumulation of verbascoside and chlorogenic acid occurred in the same organ that exhibited the highest Folin–Ciocalteu-derived TPC and the highest assay-based RACI value, as well as strong DPPH radical scavenging, CUPRAC activities, and, independently, pronounced α-glucosidase-inhibitory activity. However, this co-occurrence should not be interpreted as evidence that these two compounds quantitatively account for the elevated TPC or are responsible for the observed bioactivities. As noted above, the TPC value represents a gallic acid-equivalent Folin–Ciocalteu response, whereas the targeted LC–MS/MS analysis quantifies only predefined analytes; the two measurements therefore cannot be interpreted as a phenolic mass balance. This pattern was also reflected in the exploratory correlation analysis. Verbascoside showed strong positive associations with DPPH (r = 0.936), CUPRAC (r = 0.900), and α-glucosidase inhibition (r = 0.918), while chlorogenic acid displayed similarly strong positive associations with DPPH (r = 0.941), CUPRAC (r = 0.905), and α-glucosidase inhibition (r = 0.922). Given the limited number of organ-level observations (*n* = 4), however, these associations should be interpreted as exploratory rather than as evidence of direct causal relationships or compound-specific contributions to activity. Importantly, these parallel organ-level associations should not be interpreted as evidence of a shared mechanism between antioxidant activity and α-glucosidase inhibition. Antioxidant assays primarily reflect radical quenching, electron transfer, hydrogen atom transfer, or reducing processes, whereas α-glucosidase inhibition is an enzyme-specific phenomenon governed by inhibitor–enzyme interactions and effects on catalytic function [3,36,37]. Thus, the simultaneous occurrence of strong antioxidant responses and α-glucosidase inhibition in the root represents co-variation of mechanistically distinct bioactivities rather than evidence of a common biochemical pathway.

The antioxidant potential of these compounds is supported by their structural characteristics. Verbascoside contains caffeoyl- and hydroxytyrosol-derived catechol moieties that provide multiple hydroxyl groups capable of hydrogen and electron donation. In chlorogenic acid (5-O-caffeoylquinic acid), the carboxyl group of caffeic acid is esterified with quinic acid; however, the two phenolic hydroxyl groups of the caffeoyl catechol ring remain available as redox-active sites. Mechanistic studies indicate that these free catechol hydroxyl groups can participate in hydrogen atom transfer and other radical scavenging pathways, although esterification and the surrounding molecular environment can modulate their reactivity relative to free caffeic acid [38,39,40]. These structural characteristics are chemically consistent with antioxidant potential at the level of the individual compounds but do not demonstrate their quantitative contribution to the antioxidant activity of the crude root extract. This structural interpretation applies specifically to the antioxidant responses and should not be extrapolated to α-glucosidase inhibition, which is considered separately in Section 3.6 on the basis of enzyme-specific evidence. Nevertheless, the antioxidant performance of the crude extract cannot be attributed to verbascoside or chlorogenic acid alone, and the independent α-glucosidase inhibitory activity likewise cannot be assigned to these compounds solely on the basis of organ-level correlations. Moreover, the antioxidant response of a chemically complex extract should not be assumed to represent the simple additive contribution of its individual phenolic constituents. Co-occurring antioxidants may exhibit additive, synergistic, or antagonistic interactions depending on their relative concentrations, chemical structures, reaction kinetics, and the radical or redox system employed [41,42,43]. In particular, apparent antagonism in radical scavenging assays may arise from differences in the reaction kinetics of individual antioxidants with the assay radical and from interactions among antioxidant-derived radical species, potentially resulting in a lower measured activity than would be predicted from the individual components [42,44]. Because the present study evaluated crude organ extracts rather than isolated constituents or defined phenolic mixtures, the occurrence and magnitude of additive, synergistic, or antagonistic interactions cannot be determined from the present data.

### 3.4. Different Antioxidant Assays Reflect Different Mechanisms

The differences in organ ranking across the antioxidant assays highlight the mechanistic complementarity of these analytical approaches. DPPH and ABTS assess the ability of antioxidants to quench radical probes through reaction pathways that may involve electron transfer (ET), hydrogen atom transfer (HAT), or mixed mechanisms depending on the antioxidant structure and reaction medium. In contrast, CUPRAC and FRAP are principally reduction-based assays that evaluate the capacity of antioxidants to reduce Cu^2+^ and Fe^3+^ complexes, respectively, whereas the phosphomolybdenum assay reflects the reduction of Mo(VI) to Mo(V). Ferrous-ion chelation represents a mechanistically distinct antioxidant strategy because it depends on the ability of constituents to coordinate Fe^2+^ rather than directly reduce or quench a radical probe [45,46].

In addition to these mechanistic differences, the apparent antioxidant capacity measured by spectrophotometric assays is influenced by the physicochemical conditions of the reaction medium. Solvent composition can affect antioxidant solubility, ionization, hydrogen bonding, and reaction kinetics, whereas pH can alter the protonation state and redox behavior of phenolic constituents and thereby modify their interaction with radical or redox probes. The DPPH assay is particularly sensitive to solvent composition, pH, and reaction kinetics, while FRAP is performed under acidic conditions and therefore reflects reducing capacity within a defined low-pH reaction environment. Solvent-dependent differences have likewise been reported for ABTS, CUPRAC, and FRAP measurements [47,48,49]. Consequently, antioxidant values obtained under different solvent, pH, or other experimental conditions should not be interpreted as directly interchangeable, and the activities reported here should be considered specific to the standardized assay conditions employed.

The root exhibited the strongest activity in DPPH and in all three reduction-based assays, including CUPRAC, FRAP, and phosphomolybdenum, and consequently yielded the highest assay-based RACI value (0.54). In marked contrast, the root displayed the weakest ferrous-ion chelating activity (IC_50_ = 17.38 mg/mL), whereas the flower, leaf, and stem showed relatively similar and substantially lower IC_50_ values (5.21–6.01 mg/mL), corresponding to stronger chelating activity than that observed for the root. This divergence illustrates that high radical scavenging or reducing capacity does not necessarily translate into strong metal chelating activity. Although the root was enriched in catechol-containing chlorogenic acid and verbascoside, metal coordination is influenced not only by the presence of hydroxyl groups but also by their position, adjacent carbonyl functionalities, ionization state, reaction conditions, and the overall extract matrix.

The greater metal chelating activity of the aerial organs may partly be related to their enrichment in flavonoid aglycones with recognized metal-binding motifs. For example, quercetin contains several potential coordination sites, including the catechol B-ring and the 3-hydroxyl/4-oxo and 5-hydroxyl/4-oxo arrangements, whereas flavones such as luteolin can coordinate metal ions through catechol and 5-hydroxyl/4-oxo functionalities [50]. Nevertheless, because the present study evaluated crude extracts rather than isolated constituents, the greater chelating activity of the aerial organs cannot be assigned to individual flavonoids alone.

ABTS radical scavenging activity was comparatively similar among the four organs, particularly when expressed as Trolox equivalents, despite their markedly different phenolic profiles. In contrast, DPPH activity displayed pronounced organ dependence, with the root exhibiting approximately four-fold greater activity than the flower in Trolox-equivalent terms. The differing behavior of the extracts in the DPPH and ABTS assays may reflect differences in probe chemistry, reaction medium, reaction kinetics, and the relative reactivity of individual constituents rather than a uniform distribution of antioxidant compounds among organs.

To restrict the composite index to direct antioxidant measurements, TPC and TFC were excluded from the RACI calculation, and the index was based exclusively on the six antioxidant assay outcomes. A sensitivity comparison with an alternative formulation additionally incorporating TPC and TFC showed that root and stem retained the first and second positions, respectively, whereas the relative order of leaf and flower was reversed. This indicates that the predominance of the root was maintained after exclusion of the compositional variables, while the ranking of the lower scoring organs was more sensitive to index composition. Nevertheless, because the RACI assigns equal weight to the standardized outcomes of the included assays and several assays reflect partially overlapping antioxidant mechanisms, the index should be interpreted as a relative, descriptive measure of assay-integrated antioxidant performance rather than as an independent measure of a specific antioxidant mechanism [51].

Collectively, these assay-dependent patterns emphasize the value of employing multiple complementary methods when characterizing the antioxidant properties of chemically complex plant extracts.

### 3.5. Cholinesterase and Tyrosinase Inhibitory Activities

The four *O. bilgeri* organ extracts displayed distinct cholinesterase inhibitory profiles, although AChE and BChE inhibition was substantially weaker than that of galantamine. For AChE, the stem showed the lowest IC_50_ value, whereas the leaf was the least active organ; however, the IC_50_ values of the stem, flower, and root were relatively close in magnitude. In contrast, BChE inhibition showed a clear organ-dependent order of leaf > stem > flower > root. The corresponding AChE activities of the present extracts (2.50–3.15 mg GALAE/g extract) were comparable in magnitude to those reported for *O. vulgare* preparations by Luca et al. [17], including residual water (1.92 mg GALAE/g), spent (2.55 mg GALAE/g), and total extracts (3.27 mg GALAE/g). More recent studies further demonstrate substantial variability in cholinesterase inhibition among *Origanum* taxa and preparations. Gök et al. [52], examining five *Origanum* species over a one-year period, reported pronounced species- and collection period-dependent variation in both AChE and BChE inhibitory activities. Similarly, de Torre et al. [53] identified the ethanolic extract of *O. vulgare* subsp. *vulgare* as a potent AChE inhibitor in conjunction with detailed phytochemical characterization. More recently, Güneş et al. [54] also demonstrated enzyme-inhibitory potential in extracts and essential oils of *O. leptocladum* and *O. vulgare* subsp. *hirtum*, further emphasizing the influence of taxon and chemical composition on enzyme-inhibitory profiles. Together, these observations indicate that cholinesterase inhibition within the genus is strongly dependent on both species and phytochemical composition.

The contrasting AChE and BChE profiles of the *O. bilgeri* organs suggest that organ-dependent differences in extract composition may influence cholinesterase selectivity. Caffeic acid was particularly enriched in the stem, the organ showing the strongest AChE inhibition. Although caffeic acid has previously been demonstrated to inhibit AChE and interact with residues within the enzyme-binding gorge in purified systems [55], the present organ-level dataset does not permit a statistically generalizable assessment of its relationship with AChE inhibition. Accordingly, the observed cholinesterase activities should not be attributed to caffeic acid or any other individual constituent without further bioactivity-guided isolation and mechanistic investigation.

Tyrosinase inhibition showed a different pattern, with the stem and root exhibiting similar and stronger activities than the leaf and flower under the present assay conditions. Notably, these two organs possessed markedly different phenolic profiles: the stem was enriched in caffeic acid, protocatechuic acid, and several related phenolic acids, whereas the root was characterized by pronounced accumulation of chlorogenic acid and verbascoside. This compositional contrast suggests that similar extract-level tyrosinase inhibition can arise from chemically distinct phenolic matrices rather than from a single shared dominant constituent. Natural phenolic compounds can modulate tyrosinase through several mechanisms, including interactions within the catalytic site and, for some structures, coordination with the binuclear copper center; however, the precise mechanism is highly dependent on molecular structure and assay conditions [56].

For comparison, Luca et al. [17] reported tyrosinase inhibitory activities of 26.40–34.89 mg KAE/g for residual water, spent, and total *O. vulgare* extracts and 67.01 mg KAE/g for the corresponding essential oil. The values obtained for *O. bilgeri* in the present study (49.42–71.22 mg KAE/g extract) therefore fall within or above the range reported for these *O. vulgare* preparations. Nevertheless, direct quantitative comparisons across studies should be interpreted cautiously because differences in extraction procedures, extract composition, enzyme source, substrate concentration, and other assay conditions can substantially influence apparent inhibitory activity.

### 3.6. α-Glucosidase/α-Amylase Selectivity Profile

A distinct inhibitory profile was observed for the two carbohydrate hydrolyzing enzymes. Under the present assay conditions, all *O. bilgeri* organ extracts were weaker inhibitors of α-amylase than acarbose, with IC_50_ values of 2.17–3.23 mg/mL compared with 0.94 mg/mL for the reference inhibitor. In contrast, all four extracts exhibited lower α-glucosidase IC_50_ values (0.97–1.10 mg/mL) than acarbose (1.16 mg/mL) under the present assay conditions, with the root showing the strongest activity. Thus, *O. bilgeri* displayed a differential in vitro inhibitory profile characterized by relatively stronger α-glucosidase inhibition and weaker α-amylase inhibition.

Preferential α-glucosidase inhibition accompanied by less pronounced α-amylase inhibition has been considered a desirable profile in the screening of carbohydrate hydrolyzing enzyme inhibitors. Excessive inhibition of pancreatic α-amylase has been proposed to increase the amount of undigested carbohydrate reaching the colon and thereby contribute to gastrointestinal effects associated with extensive carbohydrate-digesting enzyme inhibition [57]. Accordingly, stronger α-glucosidase inhibition with comparatively weaker α-amylase inhibition may represent an interesting pharmacological screening profile. However, the present in vitro findings do not permit conclusions regarding gastrointestinal tolerability, therapeutic efficacy, or clinical superiority over acarbose.

A similar differential pattern has been reported in other *Origanum* taxa. The methanolic extract of the Turkish endemic *O. leptocladum* exhibited 90.10% α-glucosidase inhibition compared with 32.02% for acarbose in the same study, whereas its α-amylase inhibition was comparatively low [54]. Likewise, an ethanolic extract of *O. vulgare* from the Atacama Andean region showed an α-glucosidase IC_50_ of 7.11 µg/mL compared with 129.32 µg/mL for acarbose, while its α-amylase IC_50_ (44.71 µg/mL) was higher than that of acarbose (29.49 µg/mL) [19]. Although direct quantitative comparisons among studies are limited by differences in extraction procedures, enzyme sources, substrates, concentrations, and assay conditions, these findings indicate that preferential α-glucosidase inhibition has been observed in multiple *Origanum* taxa.

The pronounced α-glucosidase inhibition of the *O. bilgeri* root coincided with its marked enrichment in chlorogenic acid and verbascoside, both of which showed large positive Pearson correlation coefficients at the organ level with α-glucosidase inhibition. Chlorogenic acid has independently been shown to inhibit α-glucosidase, with mechanistic studies demonstrating direct enzyme interactions and mixed-type inhibitory behavior [36]. Nevertheless, because the present experiments were conducted with crude extracts and the correlation analysis was based on only four organ-level observations, these correlation patterns should be interpreted as exploratory and the α-glucosidase inhibitory activity cannot be assigned to chlorogenic acid, verbascoside, or any other individual constituent. Bioactivity-guided fractionation, purified-compound testing, and enzyme kinetic studies will be required to identify the constituents principally responsible for this activity.

### 3.7. Phytochemical–Bioactivity Correlations

Pearson correlation analysis identified several large organ-level correlation coefficients between phytochemical variables and bioactivity parameters. TPC showed large positive Pearson correlation coefficients with DPPH (r = 0.951), CUPRAC (r = 0.980), FRAP (r = 0.958), and α-glucosidase-inhibitory activity (r = 0.973), reflecting similar patterns of variation across the four investigated organ extracts. DPPH and CUPRAC also showed a large positive correlation coefficient (r = 0.992), consistent with their similar organ-dependent activity patterns. In contrast, metal chelating activity showed large negative correlation coefficients with DPPH (r = −0.933), CUPRAC (r = −0.918), and α-glucosidase inhibition (r = −0.911), consistent with the distinct organ ranking observed for metal chelation compared with the radical scavenging and reducing capacity assays. These correlations describe parallel variation across the investigated organs and should not be interpreted as evidence that antioxidant capacity and α-glucosidase inhibition are mechanistically linked. The antioxidant assays and α-glucosidase inhibition represent distinct experimental endpoints governed by different underlying chemical and biochemical processes.

Among the individual phenolic constituents, the root-enriched compounds chlorogenic acid and verbascoside showed an almost perfect positive Pearson correlation coefficient with each other (r = 0.999), reflecting their pronounced co-accumulation in the root. Both compounds also showed large positive correlation coefficients with DPPH, CUPRAC, and α-glucosidase-inhibitory activity. Verbascoside showed coefficients of r = 0.936, 0.900, and 0.918 with DPPH, CUPRAC, and α-glucosidase inhibition, respectively, whereas the corresponding coefficients for chlorogenic acid were 0.941, 0.905, and 0.922, respectively. These organ-level covariance patterns are consistent with the high antioxidant capacity and α-glucosidase-inhibitory activity of the root extract. However, because chlorogenic acid and verbascoside were strongly co-enriched in the same organ, correlation analysis alone cannot distinguish their individual contributions to the observed bioactivities. Furthermore, these parallel associations should not be interpreted as evidence of a shared mechanism between antioxidant activity and α-glucosidase inhibition. These endpoints represent mechanistically distinct processes, and their co-variation across organs indicates only a statistical association within the present dataset.

Conversely, several flavonoids enriched predominantly in the flower and leaf showed large negative organ-level correlation coefficients with reducing capacity. In particular, FRAP showed negative coefficients with apigenin (r = −0.992), taxifolin (r = −0.988), and eriodictyol (r = −0.983). These inverse organ-level covariance patterns should not be interpreted as evidence that these flavonoids lack antioxidant activity. Rather, they primarily reflect contrasting organ-level distributions: these flavonoids were concentrated in the flower and leaf, whereas FRAP and several other antioxidant measures were highest in the root and stem. Similar considerations apply to the large correlation coefficients observed among individual phenolic compounds, which may primarily reflect shared organ-specific accumulation patterns rather than direct biochemical or biosynthetic interactions.

Overall, the correlation analysis illustrates patterns of organ-level covariation between phytochemical composition and the bioactivity profiles of *O. bilgeri*, but it does not establish causal contributions of individual constituents. Because the analysis was based on only four organ-level observations (*n* = 4), even high correlation coefficients should be interpreted cautiously and should not be regarded as statistically generalizable evidence of phytochemical–bioactivity relationships. The observed covariance patterns should therefore be regarded as exploratory and hypothesis-generating, providing a basis for future bioactivity-guided fractionation, purified-compound testing, and mechanistic investigations.

### 3.8. PCA-Based Integration

Principal component analysis provided an integrated exploratory visualization of the covariance structure among the phytochemical and bioactivity variables of *O. bilgeri*. The first two components explained 50.73% and 38.49% of the total variance, accounting for 89.22% of the cumulative variance. The loading plot revealed several distinct groups of closely oriented variables, broadly consistent with the organ-level covariation patterns identified by Pearson correlation analysis.

Verbascoside and chlorogenic acid were positioned close to each other and in the same general direction as TPC, DPPH, CUPRAC, FRAP, phosphomolybdenum activity, and α-glucosidase inhibition. This multivariate arrangement is consistent with the pronounced co-accumulation of chlorogenic acid and verbascoside in the root and with the large organ-level correlation coefficients observed between these compounds and antioxidant and α-glucosidase-inhibitory parameters. This co-orientation reflects covariance among variables across the four organs and should not be interpreted as evidence of a shared mechanism between antioxidant responses and α-glucosidase inhibition. In contrast, several flavonoids enriched in the flower and leaf, including eriodictyol, apigenin, taxifolin, luteolin, quercetin, and luteolin 7-glucoside, were oriented toward a different region of the loading space, together with ABTS activity. A further grouping comprised caffeic acid, protocatechuic acid, 3,4-dihydroxyphenylacetic acid, 3- and 4-hydroxybenzoic acids, vanillin, and ferulic acid, consistent with their coordinated accumulation, particularly in the stem. These patterns illustrate that different subsets of phenolic constituents displayed distinct organ-level patterns of covariation with bioactivity parameters rather than forming a single uniform phytochemical–bioactivity axis.

The PCA organization therefore complements the Pearson correlation analysis by providing a multivariate representation of the coordinated variation between phenolic composition and biological activities. Similar use of PCA and cluster analysis has classified *O. vulgare* accessions into distinct groups according to their phenolic composition and antioxidant properties [24], illustrating the utility of multivariate approaches for exploring phytochemical diversity within *Origanum*. Nevertheless, because the present PCA was based on only four organ-level observations, it should be regarded as exploratory and descriptive rather than confirmatory. The high cumulative variance shows that the first two principal components provide a concise representation of the covariance structure in the present dataset, but this pattern should not be interpreted as independent validation of statistically generalizable or causal phytochemical–bioactivity relationships.

### 3.9. Study Significance and Novel Contributions

To the best of our knowledge, this study provides the first characterization of the non-volatile phenolic composition of *O. bilgeri* and the first systematic comparison of the phytochemical and bioactivity profiles of its flower, leaf, stem, and root. Previous investigations of this Turkish endemic species have focused predominantly on essential oil composition and associated biological activities [14,15,16]. The present findings therefore extend the phytochemical knowledge of *O. bilgeri* beyond its volatile constituents and show pronounced organ-specific patterns of chemical differentiation.

Several findings particularly contribute to the significance of this study. LC–MS/MS profiling detected 20 phenolic compounds and revealed contrasting organ-specific patterns, including rosmarinic acid-rich aerial organs and marked root enrichment in verbascoside and chlorogenic acid. The root also exhibited the highest TPC and the highest assay-based RACI value, whereas the different organs showed distinct profiles across individual antioxidant and enzyme inhibition assays. Notably, all four organ extracts exhibited lower α-glucosidase IC_50_ values than acarbose under the present assay conditions, while showing weaker α-amylase inhibition than the reference inhibitor. This differential inhibitory profile provides an additional bioactivity characteristic of *O. bilgeri* that has not previously been reported.

Finally, the integration of targeted phenolic profiling with multiple antioxidant assays, enzyme-inhibitory measurements, an assay-based RACI, Pearson correlation analysis, and PCA provided a multidimensional assessment of organ-dependent phytochemical–bioactivity patterns. Although the multivariate analyses are exploratory because they are based on four organ-level observations, they highlight distinct covariance patterns among phenolic composition and biological activities and generate testable hypotheses for subsequent fractionation and mechanistic studies. Collectively, this study provides a phytochemical and bioactivity baseline for *O. bilgeri* and identifies its individual organs, particularly the root, as chemically differentiated sources warranting further investigation.

### 3.10. Limitations

Several limitations should be considered when interpreting the present findings. First, all antioxidant and enzyme-inhibitory activities were evaluated using in vitro assays. Consequently, the results do not account for bioavailability, gastrointestinal transformation, metabolism, pharmacokinetics, or other physiological factors that may substantially influence biological efficacy in vivo. This limitation is particularly relevant to rosmarinic acid, whose in vivo exposure is influenced by absorption and extensive metabolic transformation, including conjugation and the formation of related metabolites [33,34]. Therefore, the structural antioxidant properties and in vitro reactivity discussed for rosmarinic acid should not be interpreted as direct evidence of equivalent systemic or tissue-level efficacy of the unchanged parent compound. The activities observed for the crude extracts should therefore be regarded as indicators of in vitro bioactive potential rather than evidence of therapeutic efficacy. In addition, the antioxidant assays were conducted under the standardized pH and solvent conditions specified for each method, and the effects of systematically varying these reaction conditions were not investigated. Because antioxidant responses can depend on the physicochemical assay environment, the reported activities should therefore be interpreted as condition-dependent measurements specific to the assay protocols employed.

Second, the phytochemical analysis was based on a targeted LC–MS/MS method covering 28 predefined phenolic compounds. Although this approach enabled sensitive and quantitative comparison among organs, additional non-targeted phenolic constituents and other metabolite classes may be present but were not captured by the analytical platform. Moreover, the Folin–Ciocalteu-derived TPC values, expressed as gallic acid equivalents, are analytically non-equivalent to the concentrations of individual compounds quantified by targeted LC–MS/MS and should not be interpreted as a direct phenolic mass balance. Consequently, the compounds quantified by LC–MS/MS cannot be assumed to quantitatively account for the entire Folin–Ciocalteu-reactive pool or for the observed antioxidant and enzyme-inhibitory activities of the crude extracts. Future untargeted high-resolution metabolomic analyses could therefore provide a more comprehensive characterization of the chemical diversity of *O. bilgeri*.

Third, only one extraction solvent and one extraction procedure, ultrasound-assisted ethanol extraction, were investigated. Because extraction efficiency and selectivity depend strongly on solvent composition and extraction conditions, alternative extraction systems may yield different phytochemical and bioactivity profiles. Moreover, the biological assays were performed using crude extracts without bioactivity-guided fractionation or testing of purified constituents. Accordingly, the observed activities cannot be attributed conclusively to individual phenolic compounds or specific combinations of compounds. Because defined mixtures of isolated constituents were not evaluated, potential additive, synergistic, or antagonistic interactions among co-occurring phenolic compounds could not be distinguished experimentally. Consequently, the net antioxidant responses of the crude extracts should not be interpreted as the simple sum of the activities of their individual constituents.

Fourth, enzyme inhibition was assessed on the basis of concentration–response measurements without detailed kinetic characterization. Thus, the inhibition mode and kinetic constants such as K_i_ remain unknown. Further enzyme kinetic studies, followed where appropriate by complementary molecular interaction approaches and testing of isolated compounds, will be required to clarify the mechanisms underlying the observed inhibitory activities.

Fifth, no cellular validation, cytotoxicity assessment, or safety evaluation was performed. Consequently, the biological relevance and safety of the active extracts, particularly those showing pronounced antioxidant or α-glucosidase-inhibitory activities, require confirmation in physiologically relevant cellular systems and subsequently in suitable in vivo models. Moreover, quinone-type oxidation products potentially arising from catechol-containing phenolic constituents were not directly identified, quantified, or evaluated toxicologically in the present study. Therefore, their formation and potential contribution to electrophilic reactivity, oxidative stress, or cytotoxicity remain unresolved and should be addressed in future mechanistic and safety studies.

Sixth, a single composite extract was prepared for each organ from plant material pooled across multiple individuals, and the analytical and biological activity measurements were performed in triplicate as technical replicates rather than as independent biological replicates. Consequently, the variability reported within each organ primarily reflects analytical repeatability and does not capture plant-to-plant biological variation. Technical replicates characterize measurement precision but cannot substitute for independent biological experimental units when estimating biological variability or making population-level statistical inferences [58,59]. Accordingly, the organ-level differences observed in the present study are interpreted descriptively rather than inferentially and require confirmation using independently collected and extracted biological replicates.

Seventh, the RACI was used as an equal-weight descriptive index integrating the six direct antioxidant assay outcomes. Because some of these assays reflect partially overlapping redox processes and may exhibit similar patterns of variation, the index does not explicitly account for redundancy or covariance among assay endpoints. Therefore, the RACI should be interpreted as a relative assay-integrated summary rather than as an independent or mechanistically specific measure of antioxidant capacity [51].

Eighth, the Pearson correlation and PCA analyses were based on only four organ-level observations. This limited sample size restricts the stability and generalizability of the observed multivariate patterns, and high correlation coefficients may largely reflect coordinated organ-specific distributions rather than direct biochemical relationships. These multivariate analyses should therefore be regarded as exploratory and hypothesis-generating rather than confirmatory evidence of statistically generalizable or causal phytochemical–bioactivity relationships.

Finally, the present study did not address seasonal, geographical, environmental, or population-level variation in the phytochemical composition of *O. bilgeri*. Because specialized-metabolite accumulation can vary with developmental stage, habitat, climatic conditions, and genotype, studies involving multiple populations, collection periods, and growing conditions will be necessary to determine the reproducibility and broader representativeness of the organ-specific patterns reported here.

## 4. Materials and Methods

### 4.1. Plant Material

*Origanum bilgeri* P.H. Davis was collected on 22 August 2025 from calcareous rocky habitats in the Serebel Kuyusu locality near Güzelsu (Akseki district, Antalya Province, Turkey; 37°45′56″ N, 31°49′53″ E; 1120–1170 m a.s.l.). Plant material was collected from multiple individuals within the same population. Flowers, leaves, stems, and roots were separated from the collected individuals and pooled by organ to obtain representative composite samples. Taxonomic identification was confirmed by Dr. Bedrettin Selvi, and a voucher specimen (GOPU 9198) was deposited at the Herbarium of the Faculty of Arts and Sciences, Tokat Gaziosmanpaşa University. The pooled organ samples were shade-dried at room temperature, ground into a fine powder, and stored in airtight containers until extraction.

### 4.2. Preparation of Extracts

The dried and powdered flower, leaf, stem, and root samples of *O. bilgeri* were extracted separately with HPLC-grade ethanol (Merck, Darmstadt, Germany) using ultrasound-assisted extraction (UAE). Briefly, 10 g of each powdered plant material was mixed with 200 mL of ethanol (solid-to-solvent ratio, 1:20, *w*/*v*) and extracted in an ultrasonic bath (Bandelin, Berlin, Germany) (approximately 40 kHz, 260 W) for 3 h [60]. During extraction, the vessels were externally cooled using an ice bath to maintain the extraction temperature at approximately 40°C.

Following extraction, the mixtures were filtered through Whatman No. 1 filter paper to remove plant residues. The ethanolic extracts were concentrated under reduced pressure at 40°C using a rotary evaporator (Heidolph Hei-VAP, Schwabach, Germany). The resulting dried extracts were transferred into airtight containers and stored at 4 °C until further analysis.

Extraction yields, expressed as percentages of the initial dry plant material, were 15.95%, 14.55%, 15.49%, and 16.02% for the flower, leaf, stem, and root, respectively.

### 4.3. Determination of Total Phenolic and Flavonoid Contents

The total phenolic content (TPC) of the extracts was determined using the Folin–Ciocalteu colorimetric method, as previously described by Karakus [61]. Briefly, 0.25 mL of the extract solution was mixed with diluted Folin–Ciocalteu reagent (1:9, *v*/*v*), followed by the addition of 1% Na_2_CO_3_ solution. After incubation at room temperature for 2 h, the absorbance was measured at 760 nm. Gallic acid was used for calibration, and the results were expressed as mg gallic acid equivalents (GAE)/g extract. Accordingly, the reported TPC values represent gallic acid-equivalent Folin–Ciocalteu responses and should not be interpreted as the absolute mass concentration of phenolic compounds in the extracts.

The total flavonoid content (TFC) was determined using the aluminum chloride colorimetric method, as previously described by Karakus [62]. Briefly, 1.0 mL of the extract solution was mixed with an equal volume of 2% AlCl_3_ solution. After incubation at room temperature for 10 min, the absorbance was measured at 415 nm. Rutin was used for calibration, and the results were expressed as mg rutin equivalents (RE)/g extract.

### 4.4. LC–MS/MS-Based Phytochemical Analysis

The phenolic compositions of the ethanolic flower, leaf, stem, and root extracts of *O. bilgeri* were analyzed using a previously validated liquid chromatography–electrospray ionization–tandem mass spectrometry (LC–ESI–MS/MS) method described by Cittan and Çelik [63]. A total of 28 phenolic compounds were targeted. Analyses were performed using an Agilent 1260 Infinity liquid chromatography system (Agilent Technologies, Waldbronn, Germany) coupled to an Agilent 6420 Triple Quadrupole mass spectrometer equipped with an electrospray ionization (ESI) source.

Chromatographic separation was achieved on a Poroshell 120 EC-C18 column (100 × 4.6 mm, 2.7 μm; Agilent Technologies, Santa Clara, CA, USA) using water containing 0.1% (*v*/*v*) formic acid and methanol as the mobile phases. Phenolic compounds were identified by comparison of their retention times and multiple reaction monitoring (MRM) transitions with those of authentic reference standards and quantified using external standard calibration. Quantitative results were expressed as μg/g extract.

Detailed chromatographic conditions, gradient elution program, LC–MS/MS operating parameters, MRM transitions, calibration equations, coefficients of determination (R^2^), limits of detection (LODs), and limits of quantification (LOQs) are provided in Supplementary Tables S1 and S2.

### 4.5. Antioxidant Activity Assays

The antioxidant activities of the ethanolic extracts obtained from the flower, leaf, stem, and root of *O. bilgeri* were evaluated using six complementary in vitro assays representing different antioxidant mechanisms, including phosphomolybdenum total antioxidant capacity [64], DPPH radical scavenging [65], ABTS radical scavenging [66], cupric reducing antioxidant capacity (CUPRAC) [67], ferric reducing antioxidant power (FRAP) [68], and ferrous-ion chelating activity [69], with slight modifications [70,71,72,73].

Trolox (6-hydroxy-2,5,7,8-tetramethylchroman-2-carboxylic acid) was used as the reference standard for the DPPH, ABTS, CUPRAC, FRAP, and phosphomolybdenum assays, whereas EDTA (ethylenediaminetetraacetic acid) was used as the reference standard for the ferrous-ion chelating assay. Antioxidant activities were expressed as mg Trolox equivalents (TE)/g extract or mg EDTA equivalents (EDTAE)/g extract, as appropriate. For all antioxidant assays, extract-specific blanks were included at each tested concentration to correct for intrinsic extract absorbance, color, turbidity, and other background spectrophotometric contributions. The corresponding blank absorbance was subtracted from the measured sample absorbance before calculation of antioxidant activity. Extracts were tested over a concentration range of 0.05–5 mg/mL for the phosphomolybdenum, DPPH, ABTS, CUPRAC, and FRAP assays and 1–20 mg/mL for the ferrous-ion chelating assay. Detailed experimental procedures, reagent compositions, assay conditions, and measurement parameters are provided in the Supplementary Materials.

### 4.6. Enzyme-Inhibitory Activity Assays

The enzyme-inhibitory activities of the ethanolic extracts obtained from the flower, leaf, stem, and root of *O. bilgeri* were evaluated against acetylcholinesterase (AChE), butyrylcholinesterase (BChE), tyrosinase, α-amylase, and α-glucosidase using spectrophotometric methods previously described by Karakus [62].

Acetylcholinesterase and butyrylcholinesterase inhibitory activities were determined according to Ellman-type procedures using galantamine as the reference inhibitor. Tyrosinase inhibitory activity was determined using kojic acid as the reference inhibitor, whereas acarbose was used as the reference inhibitor for the α-amylase and α-glucosidase assays.

The inhibitory activities were expressed as mg galantamine equivalents (GALAE)/g extract, mg kojic acid equivalents (KAE)/g extract, and mg acarbose equivalents (ACE)/g extract, as appropriate. For all enzyme inhibition assays, the extracts were evaluated over a concentration range of 0.05–5 mg/mL. Extract-specific blanks were prepared at each tested concentration by omitting the corresponding enzyme while retaining the extract and the remaining assay components. The absorbance of each extract-specific blank was used for background correction before calculation of percentage inhibition, thereby minimizing interference arising from the intrinsic absorbance, color, or turbidity of the crude extracts. Detailed assay protocols, reagent compositions, incubation conditions, and measurement parameters are provided in the Supplementary Materials.

### 4.7. Expression of Results and Determination of IC_50_ and EC_50_ Values

Antioxidant and enzyme-inhibitory activities were expressed both as standard-equivalent values and as IC_50_ or EC_50_ values to enable a comprehensive comparison of the biological activities of the extracts. For concentration-dependent measurements, the extracts were evaluated over a concentration range of 0.05–5 mg/mL in all antioxidant and enzyme inhibition assays, except for the ferrous-ion chelating assay, for which a concentration range of 1–20 mg/mL was used. Background-corrected absorbance values obtained using the corresponding extract-specific blanks were used for all IC_50_ and EC_50_ calculations.

For the DPPH, ABTS, and ferrous-ion chelating assays, IC_50_ (mg/mL) was defined as the extract concentration required to achieve 50% radicalvscavenging activity or 50% inhibition of ferrous-ion–ferrozine complex formation, respectively. IC_50_ values for these antioxidant assays were estimated by linear regression analysis of the corresponding background-corrected concentration–response data.

For the CUPRAC, FRAP, and phosphomolybdenum assays, EC_50_ (mg/mL) was defined as the extract concentration corresponding to an absorbance value of 0.500. EC_50_ values were estimated by linear regression analysis using background-corrected absorbance values obtained across the tested extract concentrations.

For the enzyme inhibition assays, IC_50_ (mg/mL) was defined as the extract concentration required to inhibit 50% of the corresponding enzyme activity. IC_50_ values were calculated by nonlinear regression analysis using GraphPad Prism 10.0 (GraphPad Software Inc., San Diego, CA, USA). The nonlinear regression analyses were based on the background-corrected concentration–inhibition data obtained over the tested extract concentration range.

Lower IC_50_ or EC_50_ values indicate stronger biological activity.

### 4.8. Relative Antioxidant Capacity Index (RACI)

The relative antioxidant capacity index (RACI) was calculated, following the general approach of Sun and Tanumihardjo [51], using the standard-equivalent values obtained from the six direct antioxidant assays: phosphomolybdenum, DPPH, ABTS, CUPRAC, FRAP, and ferrous-ion chelating activity. TPC and TFC were excluded from the RACI calculation because they represent compositional measurements rather than direct antioxidant assays. For each parameter, the organ-level mean value was converted to a standardized z-score using the equation *z* = (*x* − μ)/σ, where *x* is the mean value for a given organ, μ is the mean of the corresponding parameter across the four organ extracts, and σ is the corresponding population standard deviation across the organ-level means. Because higher standard-equivalent values indicated stronger antioxidant activity for all six assays, the standardized scores were used in the same direction without further inversion. The six standardized assay scores were averaged with equal weighting to obtain the RACI value for each organ.

The RACI was used as a descriptive, relative summary of assay-integrated antioxidant performance rather than as an independent measure of a single antioxidant mechanism. Because some of the included antioxidant assays may reflect partially overlapping chemical processes, assay-specific results were also considered separately when interpreting antioxidant performance. For sensitivity analysis, an alternative RACI formulation additionally incorporating TPC and TFC was recalculated using the same standardization procedure and compared with the assay-only formulation to assess whether the descriptive organ ordering was affected by index composition.

### 4.9. Data Treatment and Multivariate Analyses

A single ethanolic extract was prepared for each plant organ. All analytical and biological activity measurements were performed in triplicate as technical replicates, and the results are expressed as the mean ± standard deviation (SD). The SD therefore reflects within-sample analytical repeatability rather than biological variation among individual plants. Because independent biological extracts were not available for the individual organs, no inferential statistical tests were applied to compare the flower, leaf, stem, and root extracts or to compare the extracts with the corresponding reference standards. Organ-level differences were therefore evaluated descriptively. Technical replicates characterize measurement precision but do not constitute independent biological experimental units for population-level statistical inference [58,59]. Pearson correlation coefficients were calculated using IBM SPSS Statistics Version 22.0 (IBM Corp., Armonk, NY, USA).

Pearson correlation analysis was performed using organ-level mean values (*n* = 4) to explore patterns of covariation among total phenolic content (TPC), total flavonoid content (TFC), quantified phenolic compounds, antioxidant activities, and enzyme-inhibitory activities. For the correlation and multivariate analyses, antioxidant and enzyme-inhibitory activities were represented by their corresponding standard-equivalent values, such that higher values consistently indicated stronger activity. The Pearson correlation matrix was visualized as a hierarchically clustered heatmap using Python-based scientific computing and visualization tools. Hierarchical agglomerative clustering of both rows and columns was performed using the unweighted pair-group method with arithmetic mean (UPGMA; average linkage), with 1 − *r* used as the distance measure. Hierarchical clustering and graphical visualization were implemented using SciPy (version 1.11.4) and Matplotlib (version 3.8.0), respectively.

Principal component analysis (PCA) was performed on the Pearson correlation matrix using Python-based numerical computation tools, and the resulting variable loadings were visualized as a PC1–PC2 correlation circle plot using Matplotlib (version 3.8.0). PC1 and PC2 explained 50.73% and 38.49% of the total variance, respectively, accounting for 89.22% of the cumulative variance. Because the Pearson correlation and PCA analyses were based on only four organ-level observations, these multivariate analyses were considered exploratory and descriptive and were used primarily to visualize patterns of covariation rather than for confirmatory statistical inference or to establish causal relationships.

## 5. Conclusions

The present study revealed distinct descriptive patterns of organ-dependent phytochemical and bioactivity differentiation in the Turkish endemic *O. bilgeri*. Rosmarinic acid predominated across all organs, whereas the flower and leaf were comparatively enriched in several flavonoids and the root displayed a distinctive phenolic signature characterized by marked accumulation of verbascoside and chlorogenic acid. The root also exhibited the highest total phenolic content and the highest assay-based RACI value, while the contrasting performance observed across individual antioxidant assays emphasized the importance of using complementary methods to characterize complex plant extracts.

The organ extracts also showed distinct enzyme-inhibitory profiles. Particularly noteworthy was the differential activity against carbohydrate hydrolyzing enzymes: all four extracts exhibited lower α-glucosidase IC_50_ values (0.97–1.10 mg/mL) than acarbose (1.16 mg/mL) under the present assay conditions, while their α-amylase inhibition was weaker than that of the reference inhibitor. Pearson correlation analysis and PCA further illustrated coordinated but exploratory organ-level patterns of covariation between phenolic composition and bioactivity variables. Collectively, these findings provide the first organ-level non-volatile phenolic and bioactivity characterization of *O. bilgeri* and identify its root as a particularly distinctive source of phenolic constituents with antioxidant and α-glucosidase-inhibitory potential. Because each organ was represented by a single composite extract and the measurements were technical replicates, the observed organ-level differences should be interpreted descriptively and require confirmation using independent biological replicates. Further studies involving bioactivity-guided fractionation, purified-compound testing, enzyme kinetics, cellular validation, and in vivo assessment are required to determine the compounds responsible for these activities and their biological relevance. Given the endemic status of *O. bilgeri*, any future utilization should also consider sustainable sourcing and conservation.

## Figures and Tables

**Table 1:** Phenolic compound profile of ultrasound-assisted ethanolic extracts obtained from different organs of *O. bilgeri* determined by LC–MS/MS analysis.

No	Compounds	Flower	Leaf	Stem	Root
1	Rosmarinic acid	2776 ± 29	3236 ± 2	3093 ± 39	1500 ± 12
2	Eriodictyol	889 ± 9	906 ± 9	113 ± 1	6.51 ± 0.10
3	Apigenin	238 ± 2	220 ± 1	27.6 ± 0.1	1.90 ± 0.05
4	Taxifolin	225 ± 1	203 ± 1	11.0 ± 0.2	ND
5	Apigenin 7-glucoside	244 ± 1	120 ± 1	226 ± 1	42.4 ± 1.3
6	Caffeic acid	87.9 ± 0.1	111 ± 1	354 ± 1	83.5 ± 0.2
7	Luteolin	79.0 ± 0.3	96.8 ± 1.0	19.2 ± 0.1	ND
8	Quercetin	40.5 ± 0.1	57.7 ± 1.6	4.81 ± 0.04	ND
9	3,4-Dihydroxyphenylacetic acid	12.0 ± 0.2	33.6 ± 0.9	91.6 ± 0.2	4.55 ± 0.20
10	Protocatechuic acid	22.5 ± 0.1	30.4 ± 1.0	198 ± 2	24.4 ± 0.3
11	Luteolin 7-glucoside	57.6 ± 0.5	16.6 ± 0.3	2.94 ± 0.13	ND
12	Vanillin	7.90 ± 0.25	11.5 ± 0.5	125 ± 5	46.0 ± 0.1
13	Ferulic acid	11.3 ± 0.3	10.7 ± 0.4	38.3 ± 0.2	16.6 ± 0.2
14	p-Coumaric acid	12.9 ± 0.1	7.54 ± 0.07	18.2 ± 0.4	2.10 ± 0.02
15	Chlorogenic acid	10.4 ± 0.1	6.98 ± 0.06	28.6 ± 0.4	1461 ± 22
16	4-Hydroxybenzoic acid	9.04 ± 0.34	6.12 ± 0.03	23.8 ± 1.5	6.86 ± 0.10
17	3-Hydroxybenzoic acid	9.10 ± 0.21	6.10 ± 0.07	23.8 ± 1.0	6.63 ± 0.10
18	Hyperoside	5.35 ± 0.09	5.15 ± 0.03	7.30 ± 0.05	2.71 ± 0.13
19	Verbascoside	2.37 ± 0.03	4.02 ± 0.14	4.14 ± 0.55	2775 ± 55
20	Gallic acid	2.78 ± 0.17	2.25 ± 0.04	10.2 ± 0.7	0.57 ± 0.03
21	(−)-Epicatechin	ND	ND	ND	ND
22	(+)-Catechin	ND	ND	ND	ND
23	2-Hydroxycinnamic acid	ND	ND	ND	ND
24	Hesperidin	ND	ND	ND	ND
25	Kaempferol	ND	ND	ND	ND
26	Pinoresinol	ND	ND	ND	ND
27	Sinapic acid	ND	ND	ND	ND
28	Syringic acid	ND	ND	ND	ND

Values are expressed as the mean ± standard deviation (SD) of three technical replicate measurements for each composite organ extract and are reported as µg/g extract. The reported SD values reflect analytical repeatability rather than biological variation among individual plants. Because each organ was represented by a single composite extract, no inferential statistical comparisons were performed among organs. ND indicates compounds that were not detected under the experimental conditions. Phenolic compounds were identified and quantified by LC–MS/MS using authentic reference standards based on retention times and multiple reaction monitoring (MRM) transitions.

**Table 2:** IC_50_ and EC_50_ values (mg/mL) of antioxidant activities of ultrasound-assisted ethanolic extracts obtained from different organs of *O. bilgeri.*

Assay	Flower	Leaf	Stem	Root	Standard
Phosphomolybdenum assay (EC_50_)	0.44 ± 0.013	0.62 ± 0.004	0.44 ± 0.014	0.35 ± 0.005	0.33 ± 0.007
DPPH radical scavenging (IC_50_)	1.17 ± 0.036	1.01 ± 0.003	0.54 ± 0.018	0.28 ± 0.009	0.11 ± 0.007
ABTS radical scavenging (IC_50_)	0.29 ± 0.012	0.28 ± 0.003	0.32 ± 0.004	0.31 ± 0.018	0.10 ± 0.004
CUPRAC reducing (EC_50_)	0.96 ± 0.043	0.65 ± 0.040	0.38 ± 0.010	0.22 ± 0.003	0.16 ± 0.011
FRAP reducing (EC_50_)	0.58 ± 0.021	0.40 ± 0.015	0.19 ± 0.007	0.16 ± 0.001	0.05 ± 0.003
Metal chelating activity (IC_50_)	5.21 ± 0.439	5.43 ± 1.374	6.01 ± 0.583	17.38 ± 0.27	0.02 ± 0.001

Values are expressed as the mean ± SD of three technical replicate measurements for each composite organ extract. The reported SD values reflect analytical repeatability rather than biological variation among individual plants. Because each organ was represented by a single composite extract, no inferential statistical comparisons were performed among organs or between organ extracts and the corresponding reference standards. IC_50_ or EC_50_ values are expressed as mg/mL and lower IC_50_ or EC_50_ values indicate stronger antioxidant activity. Trolox was used as the reference standard for the phosphomolybdenum, DPPH, ABTS, CUPRAC, and FRAP assays. EDTA was used as the reference standard for the metal chelating assay.

**Table 3:** IC_50_ values (mg/mL) of enzyme-inhibitory activities of ultrasound-assisted ethanolic extracts obtained from different organs of *O. bilgeri.*

Assay	Flower	Leaf	Stem	Root	Standard
Acetylcholinesterase inhibition	1.09 ± 0.050	1.28 ± 0.019	1.02 ± 0.007	1.12 ± 0.014	0.0032 ± 0.0002
Butyrylcholinesterase inhibition	1.13 ± 0.017	0.98 ± 0.004	1.05 ± 0.029	1.24 ± 0.010	0.0032 ± 0.0002
Tyrosinase inhibition	1.67 ± 0.064	1.61 ± 0.027	1.16 ± 0.015	1.23 ± 0.002	0.08 ± 0.002
α-Amylase inhibition	2.62 ± 0.022	2.39 ± 0.021	2.17 ± 0.002	3.23 ± 0.010	0.94 ± 0.035
α-Glucosidase inhibition	1.10 ± 0.003	1.06 ± 0.002	1.05 ± 0.007	0.97 ± 0.004	1.16 ± 0.026

Values are expressed as the mean ± SD of three technical replicate measurements for each composite organ extract. The reported SD values reflect analytical repeatability rather than biological variation among individual plants. Because each organ was represented by a single composite extract, no inferential statistical comparisons were performed among organs or between organ extracts and the corresponding reference standards. IC_50_ values are expressed as mg/mL and lower IC_50_ values indicate stronger enzyme-inhibitory activity. Galantamine was used as the positive control for acetylcholinesterase and butyrylcholinesterase inhibition assays. Kojic acid was used as the reference standard for the tyrosinase inhibition assay, whereas acarbose was used as the positive control for the α-amylase and α-glucosidase inhibition assays.

## Data Availability

The original contributions presented in this study are included in the article/Supplementary Materials. Further inquiries can be directed to the corresponding author(s).

## Supplementary Materials

The following supporting information can be downloaded at https://www.mdpi.com/article/10.3390/ph19091429/s1: Table S1: ESI–MS/MS Parameters and analytical characteristics for the Analysis of Target Analytes by MRM Negative and Positive Ionization Mode; Table S2: Calibration curves and sensitivity properties of method.
